# Building consensus and identifying priorities: using a Delphi technique to define core elements and clinical determinates for a nutrition model of care for people with cancer

**DOI:** 10.1007/s11764-026-01984-9

**Published:** 2026-02-13

**Authors:** Jenelle Loeliger, Anna Ugalde, Judi Porter, Nicole Kiss

**Affiliations:** 1 Nutrition and Speech Pathology Department, Peter MacCallum Cancer Centre, 305 Grattan Street, Melbourne, VIC 3000 Australia; 2 Institute for Physical Activity and Nutrition (IPAN), Deakin University, 1 Gheringhap Street, Geelong, VIC 3220 Australia; 3 School of Exercise and Nutrition Sciences, Deakin University, 1 Gheringhap Street, Geelong, VIC 3220 Australia; 4 Sir Peter MacCallum Department of Oncology, University of Melbourne, Grattan Street, Parkville, VIC 3010 Australia; 5 Institute for Health Transformation, Deakin University, 1 Gheringhap Street, Geelong, VIC 3220 Australia

**Keywords:** Nutrition, Cancer, Delphi study, Model of care

## Abstract

**Purpose:**

Access to cancer nutrition care is often poor, and it can be challenging for cancer services to put models of care into practice. The aim of this study was to seek consensus from consumers of cancer services and nutrition experts on the core elements and key clinical determinants for a nutrition model of care for people with cancer.

**Methods:**

A two-round, national online Delphi study was conducted February–May 2025. Proposed items were presented to a national panel of nutrition experts and consumers. Agreement was rated on an 11-point Likert scale for importance and feasibility against 62 items. Response frequencies were assessed against a priori thresholds to determine consensus ratings.

**Results:**

Sixty-nine panellists participated in Round 1 (68.1% nutrition experts, 31.9% consumers) and 60 in Round 2 (68.3% nutrition experts, 31.7% consumers). Strong consensus was reached (both importance and feasibility) for 32 items that underpin cancer nutrition care. Findings identified opportunities for enhancing cancer nutrition care and improving feasibility using strategies beyond workforce and resource allocation, including elevating the value and practice of nutrition care.

**Conclusion:**

Findings will inform system-level guidance for cancer nutrition care within complex health environments.

**Implications for Cancer Survivors:**

Consensus regarding the critical elements of a nutrition model of care based on importance and feasibility, and underpinned by national expert consensus, has the potential to address systemic barriers to access and unwarranted variation in care, and bridge the evidence–practice gap.

**Supplementary information:**

The online version contains supplementary material available at https://doi.org/10.1007/s11764-026-01984-9.

## Introduction

Cancer is a growing global burden, and it is estimated that 1 in 5 people will develop cancer in their lifetime [1]. Nutrition disorders such as malnutrition, sarcopenia, and cachexia are highly prevalent and have significant physiological and psychological impacts for people with cancer [2–7]. Nutrition also plays a critical role in optimising body composition and physical health, reducing the risk of cancer recurrence and in the long-term management of cancer as a chronic disease into survivorship [8, 9]. Evidence continues to grow for nutrition interventions that optimise health-related outcomes for people with cancer, including improvements in survival, quality of life, physical function, and health care costs [2, 6, 10–13]. Despite this, we see reduced access to nutrition information and dietetic care for malnourished individuals and unwarranted variation in nutrition care across the cancer care continuum [10, 14, 15].

As a multi-faceted concept, a model of care is supported by theory, evidence, and standards, and can be defined by core elements and principles to provide a framework for implementation and evaluation [16, 17]. A number of resources exist that are relevant to cancer care and that support the development of models of care [17], quality standards [18], guidelines for chronic diseases [19, 20], frameworks describing cancer supportive care [21–23] and models of cancer survivorship [24–26]. There are also Optimal Care Pathways, which provide tumour-specific and population group standards for the delivery of safe, quality cancer care [27]. Importantly, many of these resources also help design and implement care that is comprehensive, multidisciplinary, and focused on value-based healthcare, whereby value is recognized as what matters to patients [21, 22].

A range of evidence-based guidelines [2, 8–11, 28], frameworks describing the Nutrition Care Process [29, 30] and expert recommendations [6, 12] are available to guide best practice cancer nutrition care. However, there is a distinct absence of an overarching or system-level model of care that provides a framework articulating the minimum standards and strategies for improving and supporting the delivery of cancer nutrition care in clinical practice. A recent scoping review found that internationally published cancer nutrition models of care are primarily limited to metropolitan hospital settings, commonly lack evidence-based components such as valid nutrition assessment tools, implementation frameworks, and the inclusion of people with cancer within the design [31]. The scoping review identified ten core elements that underpinned and supported the delivery of nutrition care and provided insights for the critical elements to consider for inclusion in a model of care [31]. These included timely care driven by a care pathway, protocol, or clinic; nutrition expert-led; flexible and integrated; with multi-directional communication and multidisciplinary engagement; accessible; across all time points and stratified by risk.

The development and implementation of evidence-based models of care into clinical practice are incredibly challenging [12, 32, 33]. However, the potential benefits for people with cancer and health services are only realized with the successful implementation of best practice evidence-based recommendations, including for nutrition care. There is an opportunity to develop pragmatic guidance on how to deliver optimal nutrition care to people with cancer within resource-constrained, complex health care environments [34, 35]. A deeper investigation into the views and experiences of health professionals who are responsible for planning and delivering care, and those receiving cancer nutrition care, would support an improved understanding of the most important and feasible components of care within health service settings. Building on our scoping review, expert consensus is needed on the core elements of a nutrition model of care and to help identify and prioritize the key clinical determinants (factors that influence) for a nutrition model of care in real-world clinical settings. This will improve the conceptualization and understanding of the critical elements required for an optimal nutrition model of care and the necessary clinical and policy considerations required to support the adoption of such a model in clinical care. Therefore, the aim of this study is to seek consensus from consumers of cancer services and cancer nutrition experts on the core elements and key clinical determinants for a nutrition model of care for people with cancer.

## Methods

A national two-round, online modified Delphi consensus study was conducted between February and May 2025 and undertaken in accordance with Conducting and REporting DElphi Studies (CREDES) recommendations [36]. Ethical approval for this study was obtained from Deakin University Human Research Ethics Committee (2024/HE000624).

### Study population

Eligible participants for the expert panel were identified from two key groups from within Australia: (1) cancer nutrition experts and (2) cancer consumers. Cancer nutrition experts included the following: dietitians (≥ 3 years full-time experience or pro-rata for part-time, managing/treating people with cancer in any healthcare setting) and/or any health professionals (≥ 5 years in any healthcare setting managing/treating people with cancer and working alongside a dietitian within a multidisciplinary team) and/or academics (published in the field of cancer and nutrition in the past 5 years). Cancer consumers included people with (or previously with) a diagnosis of cancer and their caregivers. In addition, all participants needed to be aged ≥ 18 years, live/work in Australia, able to read and understand English to a proficient level, and able to access the internet.

There is no recognized sample size for Delphi techniques; however, a minimum of eight participants is recommended, with greater numbers likely to produce more stable findings and diverse opinions [37, 38]. To ensure balanced breadth across expertise and professional backgrounds, and participants with experience in cancer nutrition, 30–50 total participants were set as the target for this study.

### Delphi study method

#### Panel recruitment

Participants were recruited purposively by distributing the survey invitation via key professional organisations (i.e. Clinical Oncology Society of Australia, Dietitians Australia, Dietitian Connection, The Victorian Cancer Malnutrition Collaborative) and consumer networks (i.e. Cancer Council, Cancer Voices Australia, Peter MacCallum Cancer Centre consumer register) using email, mailing lists, social media or other methods preferred by the organisations. Snowballing recruitment was utilized where organisations and potential participants were encouraged to forward or distribute the study invitation through their networks.

Invitations included a survey link administered in Research Electronic Data Capture (REDCap) [39]. A Plain Language Statement was provided electronically within the survey (including a clear description of the Delphi aims and the survey development process), and participants provided confirmation of their understanding and consent prior to completing the survey questions in REDCap. No incentives were provided to participants.

#### Generation of proposed items

Preliminary work to generate the proposed items included the following: (1) a comprehensive scoping review that identified from the international literature the core elements that underpinned and supported nutrition models of care for people with cancer in clinical practice [31]; and (2) the research team generated a list of clinical determinants related to the development and delivery of cancer nutrition care into clinical practice (under categories: staffing and resources; environment, space and technology; stakeholder engagement, awareness and support; access to care and clinical practices; training and education; nutrition governance and data; people with cancer and caregiver input). A combined list of 62 proposed items, including 10 core elements and 52 clinical determinants, was included in Round 1 of the Delphi process.

#### Delphi survey procedure

This study utilized a modified Delphi process, whereby detailed preliminary work to generate the proposed items (as described above) for the Round 1 survey was employed rather than starting with the open qualitative approach used in traditional Delphi methodology [36, 38]. Further, a two-round Delphi study was planned with a Round 3 survey proposed in the instance that no proposed items reached strong consensus within the Round 1 and/or Round 2 surveys. The Round 1 and Round 2 surveys were developed by the research team and underwent face validity and pilot testing with four potential participants (i.e. two cancer nutrition experts, two cancer consumers), with feedback incorporated into the final surveys. The surveys comprised three parts: (1) demographic questions, (2) core elements, and (3) clinical determinants (Supplementary Material 1). The survey questions were the same for all panel members, apart from the demographic questions and the use of less technical language for some questions in the cancer consumers’ survey.

### Round 1

The Round 1 survey was open for 4 weeks (10/02/2025–07/03/2025) and participants were sent two email reminders. Participants were asked to rate all 62 proposed core elements and clinical determinant items against two questions:

- *Importance*: How important do you think this element/clinical determinant is in underpinning and supporting a nutrition model of care for people with cancer?
- *Feasibility*: How feasible do you think this element/clinical determinant is to incorporate and adopt into a clinical setting/your health service?

Each question comprised an 11-point unipolar Likert scale from ‘0’ (not at all important/feasible) to ‘10’ (extremely important/feasible), or unable to rate. Participants could provide free-text comments against each item and could suggest any missing items for consideration for inclusion in the subsequent round.

### Round 2

Participants who fully or partially completed the Round 1 survey were invited to complete the Round 2 survey using a unique link; no new participants were recruited. The Round 2 survey was open for 4 weeks (29/04/2025–27/05/2025) and participants were sent two email reminders. Participants were presented with the results of Round 1 for their reference and consideration including: (1) a summary of the items that reached strong consensus (and were removed from the Round 2 survey), (2) the median score and interquartile range (IQR) from the Round 1 survey for each item retained in Round 2, and (3) each participant’s individual ratings from the Round 1 survey for each item.

### Data analysis

Descriptive statistics were used to analyse and summarise demographic data and survey responses from Round 1 and 2. Only fully completed surveys were included in the analysis (incomplete surveys excluded for the purposes of analysis). For each survey round, responses to all Likert items (for both importance and feasibility) were defined as: highly rated (Likert score ≥ 7–10), moderately rated (Likert score 4–< 7), or low rated (Likert score < 4). The total number, relative frequencies, median, and IQR were calculated for each item for both importance and feasibility. There is wide variation for agreed consensus criteria in Delphi studies; however, it is recommended that a priori thresholds are applied [40, 41]. For this study, relative frequencies for each item were assessed against a priori thresholds to determine combined consensus ratings of either *strong* (≥ 70% of participants indicated a highly rated response for both importance and feasibility), *medium* (≥ 70% of participants indicated a highly rated response for one of importance or feasibility and between 50% and < 70% indicated a highly rated response for the alternate), or *weak* consensus (< 50% of participants indicated a highly rated response for both importance and feasibility or one < 50% and the alternate < 70%) [40]. The research team convened to discuss the Round 1 survey responses and decisions regarding item inclusion for the Round 2 survey. Items with *strong* consensus were removed for subsequent rounds as they were considered to have reached consensus; items with *medium* and *weak* consensus were reviewed by the research team to decide if they would be retained or removed for the subsequent round. Following the last survey round, a final set of core elements and key clinical determinants items that reached *strong* combined consensus from all survey rounds were reported. All responses to open-ended questions were considered to support the interpretation of the quantitative results. In addition, the research team members met and assessed key points from the responses, with a focus on those relating to improving feasibility. Microsoft Excel™ was utilized for all data analysis.

## Results

### Delphi panel

Sixty-nine participants consented and fully completed the Round 1 survey (Table 1). The response rate for Round 1 could not be determined, as survey distribution was via key professional organisations/networks and snowballing was encouraged to maximise participation. A further 38 participants partially completed Round 1, resulting in 107 participants invited to participate in the Round 2 survey. Sixty participants consented and fully completed the Round 2 survey (56.1% response rate), with *n* = 50 participants completing both the Round 1 and Round 2 surveys. Overall, cancer nutrition experts made up approximately two-thirds (*n* = 47, 68.1% Round 1; *n* = 41, 68.3% Round 2) and cancer consumers one-third (*n* = 22, 31.9% Round 1; *n* = 19, 31.7% Round 2) of participants in both survey rounds.

**Table 1:** Delphi participant characteristics across round 1 and 2 surveys

Demographic variable	Round 1		Round 2	
	***n* = 69**		***n* = 60**	
	***n***	**%**	***n***	**%**
**All participants**	**69**	**100.0**	**60**	**100.0**
Role				
Cancer consumer	22	31.9	19	31.7
Person with cancer	16	23.2	11	18.3
Caregiver	6	8.7	8	13.3
Cancer nutrition expert	47	68.1	41	68.3
**Cancer consumers**	**22**	**31.9**	**19**	**31.7**
Geographic descriptor^a^				
Metropolitan area	14	63.6	11	57.9
Regional centre	6	27.3	6	31.6
Large rural town	2	9.1	1	5.3
English first language				
Yes	19	86.4	17	89.5
No	3	13.6	2	10.5
Cancer diagnosis^b^				
Breast	7	31.8	4	21.1
Head and neck	4	18.2	5	26.3
Lung	3	13.6	3	15.8
Blood/haematological	3	13.6	2	10.5
Unknown primary	2	9.1	2	10.5
Other	4	18.2	7	36.8
Received care from a dietitian				
Yes	14	63.6	13	68.4
No	8	36.4	6	31.6
**Cancer nutrition experts**	**47**	**68.1**	**41**	**68.3**
Profession				
Dietitian	36	76.6	33	80.5
Allied Health professional	4	8.5	4	9.8
Academic	3	6.4	2	4.9
Cancer medical specialist	2	4.3	1	2.4
Nurse	1	2.1	0	0.0
Other	1	2.1	1	2.4
Workplace location^b^				
Metropolitan	37	78.7	33	80.5
Regional/rural	8	17.0	8	19.5
Other^c^	2	4.3	0	0.0
Workplace practice setting^b^				
Public hospital	36	76.6	34	82.9
University	7	14.9	7	17.1
Private hospital	5	10.6	3	7.3
Private Practice	4	8.5	2	4.9
Not-for-profit or non-government organisation	3	6.4	0	0.0
Community health	1	2.1	1	2.4
Other	1	2.1	0	0.0
Experience in cancer care/research (years)				
1 to 5 years	11	23.4	7	17.1
6 to 10 years	9	19.1	8	19.5
11 to 15 years	12	25.5	11	26.8
16 to 20 years	3	6.4	3	7.3
More than 20 years	12	25.5	12	29.3

^a^Modified Monash Model Categories (https://www.health.gov.au/topics/rural-health-workforce/classifications/mmm), ^b^multiple response allowed, ^c^responded as being on extended leave or from both metropolitan and regional/rural practice settings

Cancer nutrition experts were predominantly dietitians (*n* = 36, 76.6% in Round 1; *n* = 33, 80.5% in Round 2), working in a metropolitan setting (*n* = 37, 78.7% in Round 1; *n* = 33, 80.5% in Round 2), a public hospital (*n* = 36, 76.6% in Round 1; *n* = 34, 82.9% in Round 2), represented most states and territories in Australia, and more than half had 11 years or more experience in cancer care or research (57.4% in Round 1; 63.4% in Round 2). The majority of consumer participants were people with cancer (*n* = 16/22 in Round 1; *n* = 11/19 in Round 2) rather than carers, from a metropolitan area (*n* = 14, 63.6% in Round 1; *n* = 11, 57.9% in Round 2), represented most states and territories in Australia, had experience of mixed cancer diagnoses, and over half had received care from a dietitian (*n* = 14, 63.3% in Round 1; *n* = 13, 68.4% in Round 2). No consumer participants identified as being of Aboriginal or Torres Strait Islander descent. 

### Delphi Round 1

#### Core elements

Of the 10 core elements proposed as items within the Round 1 survey: six reached *strong* consensus and were removed for the Round 2 survey and included in the final set of items; four reached *medium* consensus and were retained within the Round 2 survey; no items reached *weak* consensus; and no additional items were added nor modifications to existing items for the Round 2 survey. Of the proposed core element items that reached *medium* combined consensus, 4/4 (100.0%) had a *strong* consensus rating for importance alone and a lower rating for feasibility (either *medium* or *weak* consensus). The median, IQR, and summary of how each proposed core element item in the Round 1 survey was rated for importance and feasibility (including the combined consensus rating) is summarised in Supplementary Material 2. Most free-text comments described challenges relating to the feasibility of implementing the items into clinical practice.

#### Clinical determinants

Of the 52 clinical determinants proposed as items within the Round 1 survey: 17 reached *strong* consensus and were removed for the Round 2 survey and included in the final set of items; 34 reached *medium* consensus and one item reached *weak* consensus, with all retained within the Round 2 survey; and no additional items were added nor modifications to existing items for the Round 2 survey (Supplementary Material 2). The median, IQR, and summary of each proposed clinical determinant were rated in the Round 1 survey for importance and feasibility (including the combined consensus rating) is presented in Supplementary Material 2. The majority of free-text comments described challenges relating to the feasibility of implementing the items into clinical practice and potential strategies for overcoming barriers and enhancing feasibility.

### Delphi Round 2

#### Core elements

Of the four core elements retained within the Round 2 survey: two reached *strong* consensus and were included in the final set of items (ease of access to care; multidisciplinary engagement); and two reached *medium* consensus and were not included in the final set of consensus items (care across all time points; data integration) (Supplementary Material 2). Of the proposed core element items that did not reach *strong* combined consensus, 2/2 (100.0%) had a *strong* consensus rating for importance and a lower rating for feasibility (*medium* consensus). The median, IQR, and summary of each proposed core element rated in the Round 2 survey for importance and feasibility (including the combined consensus rating) is presented in Supplementary Material 2. Free-text comments described feasibility challenges in clinical practice.

#### Clinical determinants

Of the 35 clinical determinants retained within the Round 2 survey: seven reached *strong* consensus and were included in the final set of items; 27 reached *medium* and one reached *weak* consensus and were not included in the final set of consensus items (Supplementary Material 2). Clinical determinants that met the threshold for consensus spanned staffing and resources, environment/technology, stakeholder engagement, nutrition governance and data; and access and care practices in clinical care. Of the proposed clinical determinant items that did not reach *strong* combined consensus, 27/28 (96.4%) had a *strong* consensus rating for importance and a lower rating for feasibility (either *medium* or *weak* consensus). The median, IQR, and summary of each proposed clinical determinant rated in the Round 2 survey for importance and feasibility (including the combined consensus rating) is presented in Supplementary Material 2. Assessment of the open-ended question responses identified key opportunities to enhance feasibility and improve aspects of nutrition care beyond simply improving resource allocation. For cancer nutrition experts, this included targeted strategies such as: proactive, multidisciplinary nutrition care practices; using stratified risk approaches; digital integration and improved data utilization; advocacy and a clear value proposition for cancer nutrition care. Responses from consumers differed, where they reported they were not positioned to comment on feasibility or had assumed survey items relating to aspects of clinical practice were already part of routine cancer care and reported surprise these were not already embedded into clinical practice.

### Final consensus items

**Fig. 1:**
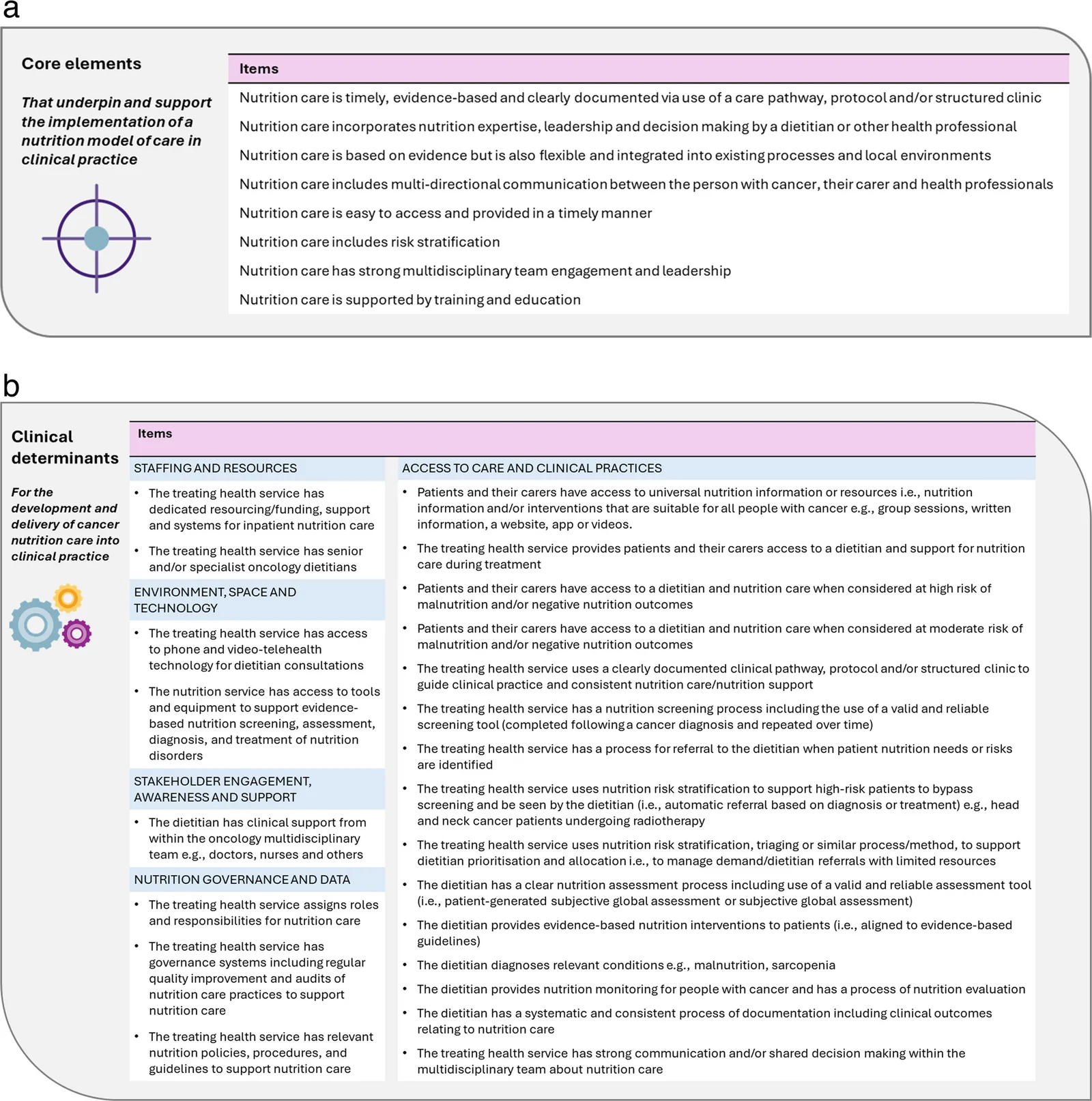
**a** Set of core element items with strong consensus for importance and feasibility. **b** Set of key clinical determinant items with strong consensus for importance and feasibility.

Overall, *strong* combined consensus was reached for both importance and feasibility for 32 items that were noted to underpin and support the implementation of a nutrition model of care for people with cancer. This included eight core elements (timely and evidence-based care; flexible care; accessible care; multidirectional communication; training and education; risk stratified care; multidisciplinary engagement; nutrition leadership) and 24 clinical determinants that underpin cancer nutrition care spanning sufficient resources; systems, governance, and qualified staff to provide evidence-based care; and infrastructure to support multidisciplinary care, triaging, and flexible access to services. Figure 1a and b indicate the final set of core element and clinical determinant items respectively, that achieved *strong* combined consensus.

## Discussion

This Delphi study has achieved consensus on a comprehensive set of core elements and key clinical determinants with high importance and feasibility in clinical practice, to support a nutrition model of care relevant to the Australian context. A total of 32 items reached consensus including eight core elements and 24 clinical determinants that underpin cancer nutrition care. This study provides new insights into aspects of nutrition clinical practice through the lens of importance and feasibility and from the combined perspectives of cancer nutrition experts (dietitians, other health professionals, academics) and cancer consumers with lived experience.

The core elements that reached consensus included key aspects of nutrition care practices, the integration of nutrition care into multidisciplinary cancer care, and reflect strong leadership, policy and governance. These core elements are well aligned to international recommendations for the delivery of high-quality nutrition and cancer care [2, 6, 19, 22, 27]. The clinical determinant items that reached consensus, spanned five of seven categories proposed in the survey (staffing and resources; environment, space and technology; stakeholder engagement, awareness and support; access to care and clinical practices; nutrition governance and data). Broadly, these items were noted as less reliant on resources/funding (i.e. dietetic workforce and space), health service stakeholder engagement and access to nutrition care across different cancer settings and timepoints.

The final set of consensus items provides direction for health services as elements of care that should be critical inclusions when designing or redesigning nutrition services for people with cancer. Further, they are highly relevant in current health service environments where it is common to see increased nutrition service demand occur alongside tight fiscal constraints. These findings also emphasise that best practice nutrition care requires consideration of the complex interplay between the realities of the modern health care environment, different settings, and the many stakeholders involved in nutrition care for people with cancer.

Greater attention to the feasibility and acceptability of nutrition care models may lead to improved implementation potential in real-world clinical settings [42]. Feasibility is crucial but often overlooked when designing and implementing models of care within complex healthcare environments [43]. Most items presented within this Delphi survey were considered highly important to panellists (10/10 for core elements; 51/52 clinical determinants) but had variable feasibility ratings. One core element item that did not reach consensus (rated highly for importance, medium for feasibility), was ‘*Nutrition care is supported across all time-points of the cancer care continuum and different settings*’. The feasibility challenges reported against this item included variable dietetic resourcing and funding models, poor communication and fragmented care transitions, and traditionally reactive nutrition care models that prioritise resourcing to admitted patients and rarely to ambulatory care settings. These challenges align with significant gaps reported within cancer services internationally, including in the United States, specifically in outpatient dietitian access and nutrition care models [44, 45].

The second core element item that did not reach consensus was ‘*Nutrition care is supported by data and data is integrated into usual processes*’*.* Despite the growing recognition and potential improvements in care possible through digital capability and integration [46], feasibility challenges were noted to be a lack of access to technology, limited resourcing, and difficulty in embedding data-driven approaches into routine care. No clinical determinant items in the categories of training/education and input from people with cancer and their caregivers reached consensus due to lower feasibility ratings.

Additional insights were gained from study participants through the Round 2 free-text responses when asked ‘*What could make this item more feasible *(*particularly considering solutions within current resources*)?’. The most frequent suggestions included clinical practice changes (i.e. proactive, multidisciplinary, risk stratified care), physical space for consultations, greater stakeholder engagement, access to education and training, digital and data-driven approaches and elevating the value of nutrition care in real-world settings. Further insight from multidisciplinary team members beyond dietitians, such as oncologists and nurses, has potential to deepen understanding of strategies required to elevate nutrition, enhance feasibility and embed nutrition practices into cancer care. Muscaritoli and colleagues have provided a useful roadmap for oncologists to embed nutrition therapy into standard clinical practice i.e. with a multidisciplinary team approach to proactively tackle common nutrition issues such as malnutrition, well-aligned with a number of the core elements that reached consensus in this Delphi study [47].

Many of the clinical determinant items that did not reach consensus, related specifically to after cancer treatment and long-term survivorship care (e.g., nutrition resourcing in primary/community care, nutrition service provision post-treatment, support for access/navigation of nutrition services between hospital and primary/community care). This is despite nutrition and diet interventions being recognized as an important aspect of high-quality cancer survivorship care internationally, and the key role it plays in optimising body composition, physical health, in chronic disease management and secondary prevention [8, 9, 19, 26]. Further work is required to better understand whether the importance and feasibility ratings relating to nutrition care after treatment/long-term survivorship is reflective of resource constraints and the difficulties in prioritising resourcing within a nutrition service, a lack of understanding of the role of nutrition at this stage or potentially for the need to explore more feasible and novel approaches across key transition points and outside of treating cancer services i.e. digital and/or community-based programs [48–51].

It is acknowledged that at a health system level, the provision of nutrition care for all people with cancer cannot always follow a one-size-fits-all approach, as nuance exists depending on the health service setting, scope, needs of the local community, and service demand. However, without a clearly articulated and comprehensive description of a minimum standard of care or framework guiding cancer nutrition care, people with cancer and their carers will continue to face inconsistent, fragmented, and reactive nutrition care, and the system will continue to remain inequitable and with unwarranted variation.

A future research opportunity lies in bridging the gap between evidence-based cancer nutrition recommendations and what occurs in clinical care; and this requires a multi-strategic approach including elevating the recognition of nutrition as a critical component of cancer, amplifying the consumer voice, defining the minimum standard to which all cancer care providers should strive toward, and providing system-level strategies to drive improvement and enable proactive, evidence-based care at a local level. This should include further investigation into possible innovative solutions for use within resource-constrained, complex health care environments including leveraging the use of innovative clinical pathways, triage tools, and risk stratification [52–54]. The set of consensus items derived from this study articulates a broad and shared understanding from cancer nutrition experts and consumers about what cancer nutrition care should look like, with consideration of both important and feasible components across the cancer care continuum. And therefore, provides an excellent foundation for future research opportunities including the development of a pragmatic cancer nutrition model of care that can help bridge this evidence to practice gap.

### Strengths and limitations

Strengths of this study was the broad stakeholder perspectives represented including a range of health professionals with expertise and knowledge of cancer nutrition care, academics and consumers (people with cancer and their carers) with a lived experience of cancer and the survey informed by a scoping review.

Limitations of this study included the length and scope of the survey, which may have challenged participants’ ability to fully engage until survey end. The recruitment approach utilized meant we were unable to determine a response rate for the Round 1 survey. Attrition between the Round 1 and 2 surveys may have impacted the Round 2 survey results, although most Round 2 survey respondents had also fully completed the Round 1 survey (83.3%) and there were no major changes to the proportional representation of cancer nutrition experts versus cancer consumers between rounds. Greater engagement from important multidisciplinary team members such as oncologists and nurses may have offered improved understanding, in particular to feasibility challenges for embedding cancer nutrition care in different settings. Furthermore, with this study being undertaken contextually within the Australian healthcare setting, the results may not be generalisable to all healthcare settings.

### Conclusion/implications for cancer survivors

Consensus on a set of core elements and key clinical determinants for a nutrition model of care was achieved by a national group of experts. Findings identified key issues relating to access, evidence-based clinical practices, leadership, workforce and resource allocation and have immediate and relevant implications for how cancer nutrition care could be equitably delivered in complex health service environments of different types, sizes, and with variable dietetic resourcing. The application of these findings will inform a system-level nutrition model of care to guide a health services’ ability to operationalise best-practice nutrition care. A nutrition model of care inclusive of the critical elements identified in this study and underpinned by national expert consensus has the potential to address systemic barriers to access and unwarranted variation in care, and ultimately enhance nutrition-related outcomes for people with cancer.

## Supplementary information

Below is the link to the electronic supplementary material.

ESM 1 (PDF 2.15 MB)

ESM 2 (PDF 309 KB)

## Data Availability

The datasets generated and analysed during the current study are available from the corresponding author on reasonable request.
