# Supplementary material for: Building consensus and identifying priorities: using a Delphi technique to define core elements and clinical determinates for a nutrition model of care for people with cancer
Source: J Cancer Surviv. 2026 Feb 13;20(Suppl 2):1543–52. doi: 10.1007/s11764-026-01984-9 (PMC13744544; doi:10.1007/s11764-026-01984-9)
Supplement: Supplementary file 1 [file 11764_2026_1984_MOESM1_ESM.pdf]

Supplementary Material 1 (ESM_1)

Building consensus and identifying priorities: using a Delphi technique to define core elements and clinical determinates for a nutrition model of care for people with cancer

J. Loeliger [a, b, c, d], A. Ugalde [e], J. Porter [b, c], N. Kiss [b, c]

[a] Nutrition and Speech Pathology Department, Peter MacCallum Cancer Centre, ,305 Grattan Street Melbourne VIC 3000, Australia

[b] Institute for Physical Activity and Nutrition (IPAN), Deakin University, 1 Gheringhap Street Geelong VIC 3220, Australia

[c] School of Exercise and Nutrition Sciences, Deakin University, 1 Gheringhap Street Geelong VIC 3220, Australia

[d] Sir Peter MacCallum Department of Oncology, University of Melbourne, Grattan Street, Parkville VIC 3010, Australia

[e] Institute for Health Transformation, Quality and Patient Safety, Deakin University, 1 Gheringhap Street Geelong VIC 3220, Australia

Corresponding author:

Jenelle Loeliger

Email: jenelle.loeliger@petermac.org

Phone: +613 8559 5161

Contents

PART A: Round 1 survey 3
PART B: Round 2 survey 23

PART A: Round 1 survey

Delphi survey - Round 1

INTRODUCTION AND PLAIN LANGUAGE STATEMENT
STUDY TITLE: Building consensus and identifying priorities: using a Delphi technique to define core elements and clinical determinants for a nutrition model of care for people with cancer<br>You are invited to participate in this research study examining the core elements and clinical determinants for a nutrition model of care for people with cancer. Our aim is to seek consensus and identify priorities from cancer consumers and cancer nutrition experts using a two-round Delphi technique.
Please download the relevant Plain Language Statement (PLS) here:<br><br>PLS cancer consumers.pdf<br><br>PLS cancer nutrition experts.pdf
Please direct any questions about this research to Jenelle Loeliger (Jenelle.Loeliger@petermac.org) or Nicole Kiss (nicole.kiss@deakin.edu.au). This study has received ethics approval at Deakin University (reference number: 2024/HE000624).

ELIGIBILITY QUESTIONS	
Have you read and understood the Plain Language Statement (PLS) attached on the previous page?	◯ Yes<br>◯ No (please read the PLS and/or direct questions to contact persons noted on the previous page)
Do you meet the eligibility criteria as outlined in the Plain Language Statement and below?<br><br>For cancer consumers:<br><br>Aged ≥18 years Live in Australia Previously diagnosed with cancer or a caregiver for someone who has Able to read and understand English to a proficient level Access to the internet to complete an online survey For cancer nutrition experts:<br><br>One or more of the following: Dietitian with ≥3 years full-time experience (or pro-rata for part-time) managing/treating people with cancer in any healthcare setting; AND/OR Any healthcare clinician or health professional with ≥5 years in any healthcare setting managing/treating people with cancer and working alongside a dietitian within a multidisciplinary team; AND/OR Researcher or academic who has published in the field of cancer and nutrition in the past 5 years.<br>Aged ≥18 years Live and work in Australia Able to read and understand English to a proficient level<br>Access to the internet to complete an online survey	◯ Yes<br>◯ No (do not proceed any further)
Do you consent to participating and wish to proceed to Round 1 of this Delphi survey?	◯ Yes<br>◯ No (do not proceed any further)

Consumers

CONTACT DETAILS. Note, this is for the purpose of inviting you to participate in the round 2 survey.

What is your FIRST name?

____

What is your SURNAME?

____

What is your email address?

____

Which group below do you belong to?

◯ Cancer consumer - person with cancer or carer of someone with cancer
◯ Cancer nutrition expert - healthcare professional and/or academic

Part 1. Demographic questions

Are you a:

◯ Person with cancer
◯ Caregiver of a person with cancer, please specify your relationship: ______

What is your postcode? To note, this helps us better identify where service gaps might be.

____

Is English your first language?

◯ Yes
◯ No, please specify your first language: ______

Do you identify as an Aboriginal or Torres Strait Islander?

◯ Yes - Aboriginal
◯ Yes - Torres Strait Islander
◯ Yes - both Aboriginal and Torres Strait Islander
◯ No
◯ Prefer not to say

Which best describes your cancer diagnosis (or the diagnosis of the person you care/cared for)?

☐ Lung
☐ Breast
☐ Genitourinary (prostate, testicular, penis)
☐ Bladder/kidney
☐ Gynaecological (cervical, ovarian, uterine, vaginal, vulvar)
☐ Head and neck
☐ Skin/melanoma
☐ Brain/spine
☐ Gastrointestinal (upper)
☐ Gastrointestinal (lower)
☐ Blood/haematological
☐ Bone or muscle/nerves (sarcoma)
☐ Unknown primary
☐ Other, please specify: ______

Did you (or the person you care/cared for) receive nutrition care from a dietitian at any point after your cancer diagnosis?

◯ Yes
◯ No
◯ Unsure

Cancer nutrition experts

Primary discipline/profession:

◯ Dietitian
◯ Academic
◯ Cancer medical specialist, please specify: ______
◯ General Practitioner
◯ Nurse
◯ Allied Health professional, please specify: ______
◯ Other, please specify: ______

State of workplace:

◯ New South Wales
◯ Victoria
◯ Queensland
◯ Western Australia
◯ South Australia
◯ Tasmania
◯ Australian Capital Territory
◯ Northern Territory

Practice setting (please select all that apply):

☐ Public hospital - metropolitan
☐ Public hospital - regional/rural
☐ Private hospital - metropolitan
☐ Private hospital - regional/rural
☐ Community health - metropolitan
☐ Community health - regional/rural
☐ Private Practice - metropolitan
☐ Private Practice - regional/rural
☐ General Practice - metropolitan
☐ General Practice - regional/rural
☐ University sector - metropolitan
☐ University sector - regional/rural
☐ Government sector - metropolitan
☐ Government sector - regional/rural
☐ Not-for-profit or non-government organisation - metropolitan
☐ Not-for-profit or non-government organisation - regional/rural
☐ Other, please specify: ______

Years of experience in cancer care/research:

◯ Less than 1 year
◯ 1-5 years
◯ 6-10 years
◯ 11-15 years
◯ 16-20 years
◯ More than 20 years

	**Part 2. Core elements of a nutrition model of care for people with cancer**
**Consumers**	We are seeking your opinion on what makes up the essential or core elements of a nutrition model of care for people with cancer. A recent scoping review of the published literature identified 10 elements from within various cancer nutrition models of care.<br><br>We would like your thoughts on (1) how important each of the proposed elements are to support a cancer nutrition model of care, and (2) how feasible you think the element is to have incorportated or put into care within a health service, based on your experience. You will be asked to rate importance on an 11-point Likert scale with 0 = not at all important, through to 10 = extremely important. Feasibility will be rated similarly on an 11-point Likert scale with 0 = not at all feasible, through to 10 = extremely feasible. You can also make comments about the elements proposed and describe any additional elements you consider to be important.<br><br>From these survey questions, we are aiming to gain expert opinion from across Australia to come up with a set of core elements that supports best practice nutrition care for people with cancer in Australia.
**Cancer nutrition experts**	We are seeking your opinion on what makes up the essential or core elements of a nutrition model of care for people with cancer. A recent scoping review of the published literature identified 10 elements that underpinned cancer nutrition models of care.<br><br>We would like your thoughts on (1) how important each of the proposed elements are in underpinning a cancer nutrition model of care in clinical practice, and (2) how feasible you think the proposed element is to implement into clinical practice, based on your experience and expertise. You will be asked to rate importance on an 11-point Likert scale with 0 = not at all important, through to 10 = extremely important. Feasibility will be rated similarly on an 11-point Likert scale with 0 = not at all feasible, through to 10 = extremely feasible. You can also make comments about each of the elements proposed and describe any additional elements you consider to be important.<br><br>From these survey questions, we are aiming to gain expert opinion from across Australia to define a set of core elements that supports best practice nutrition care for people with cancer in Australia.

CORE ELEMENT 1

Consumers

CORE ELEMENT 1. Nutrition care is timely, evidence-based and clearly documented via use of a care pathway, protocol and/or structured clinic.

Nutrition models of care are described in a format using a care pathway, protocol, structured clinic or similar, that clearly defines and documents nutrition care processes and supports timely and frequent nutrition care. These pathways, protocols or clinic models are used to support putting evidence-based care and recommendations from research into healthcare settings treating people with cancer.

Additional information/examples: The pathway, protocol or clinic model clearly describes what type of care (e.g., 1:1 face-to-face care to head and neck cancer patients), who delivers the care (e.g., nutrition screening by a nurse, nutrition assessment and management by a dietitian), where care is delivered (e.g., in an outpatient clinic in the hospital) and when care is delivered (e.g., during and after radiotherapy).

Cancer nutrition experts

CORE ELEMENT 1. Nutrition care is timely, evidence-based and clearly documented via use of a care pathway, protocol and/or structured clinic.

Nutrition models of care are described in a format using a care pathway, protocol, structured clinic or similar, that clearly and comprehensively defines and documents nutrition care processes and supports the provision of timely and frequent nutrition care. Such pathways, protocols or clinic models are used to support the implementation of evidence-based care and recommendations from research into clinical healthcare settings treating people with cancer.

Additional information/examples: The pathway, protocol or clinic model clearly describes what type of care (e.g., 1:1 face-to-face care to head and neck cancer patients), who delivers the care (e.g., nutrition screening by a nurse, nutrition assessment and management by a dietitian), where care is delivered (e.g., in an outpatient clinic in the hospital) and when care is delivered (e.g., during and after radiotherapy). In addition, the model specifies the screening and assessment tools to be used, who uses them and when, and supports decision making around nutrition care and interventions.

Please rate on the following scale: 0 = not at all important/feasible, to 10 = extremely important/feasible

	0	1	2	3	4	5	6	7	8	9	10	Unable to rate
How IMPORTANT do you think this element is in underpinning and supporting a nutrition model of care for people with cancer?	○	○	○	○	○	○	○	○	○	○	○	○
How FEASIBLE do you think this element is to incorporate and adopt into a clinical setting/your health service?	○	○	○	○	○	○	○	○	○	○	○	○

Please share any reasons for your answer or other comments about this item:

CORE ELEMENT 2

Consumers

CORE ELEMENT 2. Nutrition care incorporates nutrition expertise, leadership and decision making by a dietitian or other health professional.

Experts in diet and nutrition such as dietitians (and other health professionals such as medical specialists and nurses) play a leadership role for nutrition care. This may include dietitian-led models, or programs with more than one type of health professional including a nutrition oncology expert. Nutrition care is supported by a wide multidisciplinary team, with the nutrition expert guiding nutrition care and decision making. Nutrition experts are included in models of care that support people with high nutrition risk cancers, such as head and neck, gastrointestinal, and lung cancers, and people undergoing treatments that commonly lead to nutrition issues.

Additional information/examples: Dietitian-led or joint multidisciplinary clinics for head and neck cancer patients undergoing treatment.

Cancer nutrition experts

CORE ELEMENT 2. Nutrition care incorporates nutrition expertise, leadership and decision making by a dietitian or other health professional.

Experts in diet and nutrition such as dietitians (and other health professionals such as medical specialists and nurses) play a leadership role in the development and implementation of nutrition care processes and interventions within a nutrition model of care. This may include dietitian-led models or multimodal programs of care including a nutrition oncology expert. Nutrition care is supported by a wide multidisciplinary team, with the nutrition expert guiding, delegating and supporting best practice nutrition care and decision making in a clinical setting. Nutrition experts are included in models of care that support people with high nutrition risk cancers, such as head and neck, gastrointestinal, and lung cancers, and people undergoing treatments that commonly lead to nutrition issues.

Additional information/examples: dietitian-led or joint multidisciplinary clinics for head and neck cancer patients undergoing treatment; models incorporating nutrition expert-led decision making about starting and ceasing enteral and/or parenteral nutrition alongside other multidisciplinary team members; models incorporating a nutrition expert assessing nutritional status and diagnosing clinical conditions such as malnutrition and sarcopenia.

Please rate on the following scale: 0 = not at all important/feasible, to 10 = extremely important/feasible

	0	1	2	3	4	5	6	7	8	9	10	Unable to rate
How IMPORTANT do you think this element is in underpinning and supporting a nutrition model of care for people with cancer?	○	○	○	○	○	○	○	○	○	○	○	○
How FEASIBLE do you think this element is to incorporate and adopt into a clinical setting/your health service?	○	○	○	○	○	○	○	○	○	○	○	○

Please share any reasons for your answer or other comments about this item:

CORE ELEMENT 3

Consumers

CORE ELEMENT 3. Nutrition care is based on evidence but is also flexible and integrated into existing processes and local environments.

Models of care that are flexible and adapted to the local setting, where there has been consideration of existing processes such as team meetings, space/setup in outpatients, nutrition screening and assessment tools already utilised, referral processes, equipment and more; and these may influence the best fit of a nutrition model of care in a real-world setting.

Additional information/examples: adding on pre-treatment nutrition screening and care from a dietitian (prehabilitation) to an existing post-bowel cancer surgery model; models that create new and/or joint dietitian and multidisciplinary team consultations in the same location, that were previously medical only.

Cancer nutrition experts

CORE ELEMENT 3. Nutrition care is based on evidence but is also flexible and integrated into existing processes and local environments.

Models of care that are flexible and adapted to the local setting, where there has been consideration of existing multidisciplinary team processes such as team meetings, ward rounds and standard follow-up protocols, space in outpatients/clinics/healthcare settings, nutrition screening and assessment tools already utilised, referral practices, equipment and more; and these may influence the best fit of a nutrition model of care in a real-world setting.

Additional information/examples: models for post-operative care incorporating the introduction of early/pre-treatment nutrition screening and dietitian interventions/prehabilitation prior to gastrointestinal surgery; models that create new co-located and/or joint dietitian and multidisciplinary team consultations, clinics and follow-up arrangements that were previously medical only; models that extend existing services to increase the frequency of inpatient and outpatient dietitian review for high risk or malnourished patients following a stem cell transplant; replacement of existing consults with another health professional such as a dietitian instead of a radiation oncologist for one consult after head and neck radiotherapy or an inpatient review delegated by a dietitian to an allied health assistant.

Please rate on the following scale: 0 = not at all important/feasible, to 10 = extremely important/feasible

	0	1	2	3	4	5	6	7	8	9	10	Unable to rate
How IMPORTANT do you think this element is in underpinning and supporting a nutrition model of care for people with cancer?	○	○	○	○	○	○	○	○	○	○	○	○
How FEASIBLE do you think this element is to incorporate and adopt into a clinical setting/your health service?	○	○	○	○	○	○	○	○	○	○	○	○

Please share any reasons for your answer or other comments about this item:

CORE ELEMENT 4

Consumers

CORE ELEMENT 4. Nutrition care includes multi-directional communication between the person with cancer, their carer and health professionals.

Nutrition models of care include strong two-way communication between the person with cancer/their carer and relevant health professionals to help understanding, coordination and the delivery of care.

Additional information/examples: models that support patients to receive early nutrition therapy, education, and/or goal setting (i.e., prehabilitation) before treatment starts; models that have been developed through partnerships between health professionals and people with cancer and their carers and/or meaningful feedback is used to improve existing nutrition care.

Cancer nutrition experts

CORE ELEMENT 4. Nutrition care includes multi-directional communication between the person with cancer, their carer and health professionals.

Nutrition models of care include strong two-way communication between the person with cancer/their carer and relevant health professionals to aid understanding, coordination, and care provision.

Additional information/examples: models that support patients receiving early and proactive nutrition therapy, education, and/or goal setting (i.e., prehabilitation) before treatment starts; models that support the timely provision of tailored nutrition education material, care plans and self-management resources for people with cancer and their carers; models that have been developed in partnership with people with cancer and their carers and/or meaningful feedback is integrated into re-designing or improving existing nutrition models of care.

Please rate on the following scale: 0 = not at all important/feasible, to 10 = extremely important/feasible

	0	1	2	3	4	5	6	7	8	9	10	Unable to rate
How IMPORTANT do you think this element is in underpinning and supporting a nutrition model of care for people with cancer?	○	○	○	○	○	○	○	○	○	○	○	○
How FEASIBLE do you think this element is to incorporate and adopt into a clinical setting/your health service?	○	○	○	○	○	○	○	○	○	○	○	○

Please share any reasons for your answer or other comments about this item:

CORE ELEMENT 5

Consumers

CORE ELEMENT 5. Nutrition care is easy to access and provided in a timely manner.

Nutrition models of care are developed to support access to expert nutrition care for people with cancer. They are easy, timely, flexible and offer choice in the type of care e.g., face-to-face or telehealth. Models support people with cancer to have access to the right nutrition care at the right time; and broadly raise awareness about the importance of nutrition, where to access evidence-based nutrition information, and the nutrition services available to them.

Additional information/examples: models with quick and easy nutrition screening processes to identify nutrition risk and nutrition information proactively provided to people with cancer; models that incorporate phone or video-telehealth consultations as an option, standard care or as an alternative to face-to-face care for patient and carer convenience.

Cancer nutrition experts

CORE ELEMENT 5. Nutrition care is easy to access and provided in a timely manner.

Nutrition models of care are developed to support access to expert nutrition care for people with cancer. They are easy, proactive, timely, flexible and offer choice in different modes of care e.g., face-to-face or telehealth. Models support people with cancer to have access to the right nutrition care at the right time and broadly raise awareness about the importance of nutrition, where to access evidence-based nutrition information and the nutrition services available to them.

Additional information/examples: models that incorporate the use of blanket/automatic criteria and referrals to the dietitian for high nutrition risk groups; models with clear nutrition screening processes to identify nutrition risk and nutrition information proactively provided to people with cancer; models that incorporate phone or video-telehealth consultations as an option, standard care or as an alternative to face-to-face care for patient and carer convenience.

Please rate on the following scale: 0 = not at all important/feasible, to 10 = extremely important/feasible

	0	1	2	3	4	5	6	7	8	9	10	Unable to rate
How IMPORTANT do you think this element is in underpinning and supporting a nutrition model of care for people with cancer?	○	○	○	○	○	○	○	○	○	○	○	○
How FEASIBLE do you think this element is to incorporate and adopt into a clinical setting/your health service?	○	○	○	○	○	○	○	○	○	○	○	○

Please share any reasons for your answer or other comments about this item:

CORE ELEMENT 6

Consumers

CORE ELEMENT 6. Nutrition care includes risk stratification.

Nutrition risk stratification is included as a part of nutrition screening, nutrition assessment processes and nutrition care decision making. This includes the use of a structured approach to assign risk levels to people with cancer to support care for those at highest risk.

Additional information/examples: models that support assigning risk after nutrition screening e.g., low risk patients receive nutrition information, moderate risk patients receive care from an allied health assistant, high risk patients receive care from a dietitian.

Cancer nutrition experts

CORE ELEMENT 6. Nutrition care includes risk stratification.

Nutrition risk stratification is included as a component of nutrition screening, nutrition assessment processes and nutrition intervention decision making. This includes the use of a structured, algorithmic approach to systematically assign risk levels to people with cancer to support access and resources to those at high risk and to assign the most appropriate level of nutrition care and interventions to all people with cancer.

Additional information/examples: models that support risk stratification following completion of a nutrition screening process using a valid nutrition screening tool or specified criteria e.g., low risk patients receive nutrition information, moderate risk patients receive care from an allied health assistant, high risk patients receive care from a dietitian; models that support risk stratification and/or stepped care following a dietitian assessment i.e., meeting certain criteria determines referral for a prophylactic enteral feeding tube before treatment for head and neck cancer or meeting certain criteria such as a diagnosis of mild/moderate malnutrition to initiate commencement of oral nutrition support and increased frequency of review by the dietitian.

Please rate on the following scale: 0 = not at all important/feasible, to 10 = extremely important/feasible

	0	1	2	3	4	5	6	7	8	9	10	Unable to rate
How IMPORTANT do you think this element is in underpinning and supporting a nutrition model of care for people with cancer?	○	○	○	○	○	○	○	○	○	○	○	○
How FEASIBLE do you think this element is to incorporate and adopt into a clinical setting/your health service?	○	○	○	○	○	○	○	○	○	○	○	○

Please share any reasons for your answer or other comments about this item:

CORE ELEMENT 7

Consumers

CORE ELEMENT 7. Nutrition care has strong multidisciplinary team engagement and leadership.

Strong multidisciplinary teamwork including nutrition expert leadership, is used before, during and after a nutrition model of care goes into clinical practice. Leadership and support are gained from a range of decision makers in the health service when developing and putting a nutrition model of care into place.

Additional information/examples: the multidisciplinary team has input into developing the model and part of decision making around nutrition care; models that are new and reasonable options for care are explored and if they work, they are supported by the wider team.

Cancer nutrition experts

CORE ELEMENT 7. Nutrition care has strong multidisciplinary team engagement and leadership.

Before, during and after implementation of a nutrition model of care, strong multidisciplinary team engagement including nutrition expert leadership, is embedded within the team environment. Clinical, operational, and executive leadership and support for evidence-based nutrition care is gained for the development and implementation of nutrition models of care into clinical practice.

Additional information/examples: the dietitian and nutrition care is embedded into the multidisciplinary team processes such as within team meetings, ward rounds and multimodal programs of care; models should have the support of the multidisciplinary team through input into developing and refining the model and be embedded into clinical decision making around nutrition care; models that are novel, pragmatic, feasible and appropriate to the setting are explored and when demonstrated to be feasible, supported by the multidisciplinary team and key decision makers.

Please rate on the following scale: 0 = not at all important/feasible, to 10 = extremely important/feasible

	0	1	2	3	4	5	6	7	8	9	10	Unable to rate
How IMPORTANT do you think this element is in underpinning and supporting a nutrition model of care for people with cancer?	○	○	○	○	○	○	○	○	○	○	○	○
How FEASIBLE do you think this element is to incorporate and adopt into a clinical setting/your health service?	○	○	○	○	○	○	○	○	○	○	○	○

Please share any reasons for your answer or other comments about this item:

CORE ELEMENT 8

Consumers

CORE ELEMENT 8. Nutrition care is supported across all time-points of the cancer care continuum and different settings.

Nutrition models of care support the best nutrition care across all time-points in the cancer care pathway including before, during and after treatment. In addition, models support nutrition care in more than one setting within a single health service (i.e., between inpatients and outpatients) and between different settings and providers (i.e., between hospitals, between private/public providers, between hospitals/primary and community care).

Additional information/examples: models that provide nutrition care before, during and after treatment across both inpatients and outpatients e.g., dietitian sees patient in hospital outpatient clinic before surgery, during a hospital stay, after going home in a hospital outpatient clinic, longer-term either in a hospital outpatient clinic and/or community health; models that support transitions of care between hospital and home e.g., a regional patient is seen by dietitian after surgery as an inpatient at a city hospital and then referred to a dietitian located closer to home in primary/community care for longer-term nutrition care.

Cancer nutrition experts

CORE ELEMENT 8. Nutrition care is supported across all time-points of the cancer care continuum and different settings.

Nutrition models of care embed patient-centred and appropriate nutrition care across all time-points in the cancer care continuum (before, during and after treatment), adapted to the individual and the setting. In addition, models support nutrition care in more than one setting within a single health service (i.e., between inpatients and outpatients) and between different settings, providers and sectors (i.e., between hospitals, between private/public providers, between acute/primary and community care).

Additional information/examples: models that provide nutrition care before, during and after treatment into survivorship across both inpatients and outpatients e.g., dietitian sees patient in hospital outpatient clinic before stem cell transplant, during their inpatient admission, in hospital outpatient clinic after discharge, longer-term as needed in hospital outpatient clinic and/or community/primary care; models that support transitions of care between hospital and home e.g., a regional patient is seen by dietitian after surgery as an inpatient and in outpatient clinic during radiotherapy at a metropolitan hospital and then referred to a dietitian located closer to home in community/primary care for further post-treatment nutrition care or a shared-care arrangement between metropolitan and regional providers.

Please rate on the following scale: 0 = not at all important/feasible, to 10 = extremely important/feasible

	0	1	2	3	4	5	6	7	8	9	10	Unable to rate
How IMPORTANT do you think this element is in underpinning and supporting a nutrition model of care for people with cancer?	○	○	○	○	○	○	○	○	○	○	○	○
How FEASIBLE do you think this element is to incorporate and adopt into a clinical setting/your health service?	○	○	○	○	○	○	○	○	○	○	○	○

Please share any reasons for your answer or other comments about this item:

CORE ELEMENT 9

Consumers

CORE ELEMENT 9. Nutrition care is supported by training and education.

Nutrition experts and all staff involved in nutrition care complete specific training and education regularly. Different types of communication and awareness-raising about nutrition care is completed across the team, especially when changes to care are being made. Training for using different technology and equipment is considered for people with cancer, carers and health professionals.

Additional information/examples: models that have training for people with cancer, carers and health professions to use technology like telehealth or patient portals/access to an electronic medical record; before changing a nutrition care model, a hospital-wide presentation and training for dietitians, nurses, doctors and support workers is completed to increase awareness, understanding and knowledge about nutrition care.

Cancer nutrition experts

CORE ELEMENT 9. Nutrition care is supported by training and education.

Nutrition experts, multidisciplinary health professionals and support staff involved directly or indirectly with nutrition care undergo targeted training and education regularly and appropriate to the discipline and setting. Multi-faceted communication and awareness-raising about nutrition care is embedded within the team environment before, during and after implementation of a nutrition model of care. Training and education regarding the use of technology and/or equipment is considered for people with cancer, carers and health professionals.

Additional information/examples: models that support nurses to conduct regular training on nutrition risk screening methods and dietitians to conduct regular training on nutrition assessment methods and the diagnosis of malnutrition; models that embed appropriate training for people with cancer, carers and health professions to use equipment and technology like telehealth or patient portals/access to an electronic medical record; prior to implementation of a new model, a hospital-wide presentation and targeted training for dietitians, nurses, doctors and support workers is completed to raise awareness, understanding and knowledge about nutrition care and aid implementation.

Please rate on the following scale: 0 = not at all important/feasible, to 10 = extremely important/feasible

	0	1	2	3	4	5	6	7	8	9	10	Unable to rate
How IMPORTANT do you think this element is in underpinning and supporting a nutrition model of care for people with cancer?	○	○	○	○	○	○	○	○	○	○	○	○
How FEASIBLE do you think this element is to incorporate and adopt into a clinical setting/your health service?	○	○	○	○	○	○	○	○	○	○	○	○

Please share any reasons for your answer or other comments about this item:

CORE ELEMENT 10

Consumers

CORE ELEMENT 10. Nutrition care is supported by data and data is integrated into usual processes.

Models of nutrition care are supported by and use data appropriate to the setting. Data is used from the electronic medical record (EMR) or other data systems and from audits to improve information sharing across multidisciplinary teams and people with cancer/their carers.

Additional information/examples: models can include sharing of key information between a dietitian and patient/carer before or after a consultation; models can be monitored through audits or data that helps guide improvements to care.

Cancer nutrition experts

CORE ELEMENT 10. Nutrition care is supported by data and data is integrated into usual processes.

Models of nutrition care are supported by and utilise data appropriate to the setting. Data integration via an electronic medical record (EMR) or alternative robust data system are utilised to improve real-time operational workflows, incorporate audit and feedback within a quality improvement system and to aid information sharing across multidisciplinary teams and with people with cancer and their carers.

Additional information/examples: models can be supported by an EMR to generate automatic referrals to the dietitian, data fields for key patient-reported and clinical outcomes i.e., risk status, malnutrition diagnosis; models can include sharing of meaningful data and information with patients/carers before or after a dietitian consultation; models can incorporate dashboards/databases/nutrition data reported in a multidisciplinary meeting from an EMR/other data system to highlight nutrition risk and facilitate nutrition interventions; models can be monitored in real-time through the use of key performance indicators or audits that guide targeted care improvements.

Please rate on the following scale: 0 = not at all important/feasible, to 10 = extremely important/feasible

	0	1	2	3	4	5	6	7	8	9	10	Unable to rate
How IMPORTANT do you think this element is in underpinning and supporting a nutrition model of care for people with cancer?	○	○	○	○	○	○	○	○	○	○	○	○
How FEASIBLE do you think this element is to incorporate and adopt into a clinical setting/your health service?	○	○	○	○	○	○	○	○	○	○	○	○

Please share any reasons for your answer or other comments about this item:

GENERAL COMMENTS ABOUT CORE ELEMENTS

Are there any additional core/essential elements you consider important to underpin and support a cancer nutrition model of care?

Part 3. Key clinical determinants of cancer nutrition care

Consumers

We are seeking your opinion about the key clinical determinants for (or factors that influence and/or concerns that need to be considered), the successful implementation of cancer nutrition care into clinical practice.

The following list of items have been made by the research team. We would like your thoughts on how important each of the proposed items are as a determinant for (or a factor that influences) the implementation of cancer nutrition care into clinical care in Australia. We would also like your thoughts on how feasible you think the item is to put into place into clinical care, based on your experience. You will be asked to rate importance on a 11-point Likert scale with 0 = not at all important, through to 10 = extremely important. Feasibility will be rated similarly on a 11-point Likert scale with 0 = not at all feasible, through to 10 = extremely feasible. You can provide reasons or comments for each item. You can also describe any additional items you consider to be important.

This work is aiming to identify the most important factors and/or concerns that need to be thought about and addressed to support the implementation of the best possible models of nutrition care for people with cancer Australia-wide.

Cancer nutrition experts

We are seeking your opinion about the key clinical determinants for (or factors that influence and/or concerns that need to be considered), the successful implementation of cancer nutrition care into clinical practice.

The following list of proposed items have been generated by the research team. We would like your thoughts on how important each of the proposed items are as a determinant for (or a factor that influences) the implementation of cancer nutrition care into clinical practice in Australia. We would also like your thoughts on how feasible you think the proposed item is to implement into clinical practice, based on your experience and expertise. You will be asked to rate importance on a 11-point Likert scale with 0 = not at all important, through to 10 = extremely important. Feasibility will be rated similarly on a 11-point Likert scale with 0 = not at all feasible, through to 10 = extremely feasible. You can provide reasons or comments for each item. You can also describe any additional items you consider to be important.

This work is aiming to identify and prioritise the most important factors and/or concerns that need to be considered and addressed to support the implementation of optimal and equitable models of nutrition care for people with cancer across healthcare settings in Australia.

Please rate on the following scale: 0 = not at all important/feasible, to 10 = extremely important/feasible.

	0	1	2	3	4	5	6	7	8	9	10	Unable to rate
How IMPORTANT do you think this proposed item is as a determinant for (or a factor that influences) the implementation of cancer nutrition care into clinical practice in Australia?	○	○	○	○	○	○	○	○	○	○	○	○
How FEASIBLE do you think this proposed item is to implement into clinical practice/your health service?	○	○	○	○	○	○	○	○	○	○	○	○

Please share any reasons for your answer or comments about this item:

Proposed items

STAFFING AND RESOURCES	
1	The treating health service has dedicated resourcing/funding and availability of dietitians working in oncology
2	The treating health service has dedicated resourcing/funding, support and systems for *outpatient* nutrition clinics and care
3	The treating health service has dedicated resourcing/funding, support and systems for *inpatient* nutrition care
4	The treating health service has a stable dietitian workforce with manageable levels of staff turnover
5	The treating health service has senior and/or specialist oncology dietitians
6	The treating health service supports the dietitian to have enough time to provide nutrition care to patients (both inpatients and outpatients) and supports the development and monitoring of care models
7	In primary and community care, there are resources for and availability of dietitians providing nutrition care to people with cancer
8	Primary and community care has dietitians who have experience in providing cancer nutrition care to people with cancer

ENVIRONMENT, SPACE AND TECHNOLOGY	
9	The treating health service has a physical space for the dietitian to see patients in the outpatient environment

10	The treating health service has access to phone and video-telehealth technology for dietitian consultations
11	The treating health service has clinic space for the dietitian with the multidisciplinary team in the same area/location (i.e., co-located)
12	The nutrition service has access to tools and equipment to support evidence-based nutrition screening, assessment, diagnosis, and treatment of nutrition disorders
**STAKEHOLDER ENGAGMENT, AWARENESS AND SUPPORT**	
13	The nutrition service has buy-in and support from key decision makers in the health service
14	The dietitian has clinical support from within the oncology multidisciplinary team e.g., doctors, nurses and others
**ACESS TO CARE AND CLINICAL PRACTICES**	
15	Patients and their carers have access to free or low-cost nutrition services i.e., part of standard cancer care
16	Patients and their carers have access to universal nutrition information or resources i.e., nutrition information and/or care that is suitable for all people with cancer e.g., group sessions, written information, a website, app or videos.
17	The treating health service provides patients and their carers access to a dietitian and support for nutrition care *between diagnosis and starting treatment* (i.e., pre-surgery, pre-chemotherapy, pre-radiotherapy)
18	The treating health service provides patients and their carers access to a dietitian and support for nutrition care *during treatment*
19	The treating health service provides patients and their carers access to a dietitian and support for nutrition care *up to 3 months after treatment*
20	The treating health service provides patients and their carers access to a dietitian and support for nutrition care *>3 months post treatment* (i.e., long-term survivorship including living with advanced cancer)
21	The treating health service provides patients and their carers access and directions for nutrition care between health providers

	and/or settings (i.e., from one hospital to another, public to private provider or vice versa, from hospital to primary or community care provider)
22	Patients and their carers have access to a dietitian and support for nutrition care in primary and community care as needed
23	Patients and their carers have access to a dietitian and nutrition care when considered at *high* risk of malnutrition and/or poor nutrition
24	Patients and their carers have access to a dietitian and nutrition care when considered at *moderate* risk of malnutrition and/or poor nutrition
25	Patients and their carers have access to a dietitian and nutrition care when considered at *low* risk of malnutrition and/or poor nutrition
26	The treating health service uses a clearly documented clinical pathway, protocol and/or structured clinic to guide nutrition care
27	The treating health service has a system for the early identification of nutrition needs for people after a cancer diagnosis
28	The treating health service has a nutrition screening process including the use of a reliable screening tool (completed after a cancer diagnosis and repeated over time)
29	The treating health service has a process for referral to the dietitian when patient nutrition needs or risks are identified
30	The treating health service uses a process to support high-risk patients to automatically be seen by the dietitian e.g., head and neck cancer patients undergoing radiotherapy
31	The treating health service uses a process to support the dietitian to manage patient referrals with limited resources
32	There is a process for patients and their carers who are not able to be seen by a dietitian to receive nutrition information and care i.e., referral to community services, directed to online resources or programs
33	The treating health service uses a system where nutrition care for patients with malnutrition and sarcopenia (or other nutrition

	disorders) will be seen before patients with issues that could lead to long term poor health e.g., weight (fat) gain, heart issues
34	The dietitian has a clear nutrition assessment process including use of a reliable assessment tool (i.e., patient-generated subjective global assessment or subjective global assessment)
35	The dietitian provides evidence-based nutrition interventions to patients (i.e., aligned to the evidence and latest research)
36	The dietitian diagnoses relevant conditions e.g., malnutrition, sarcopenia
37	The dietitian monitors people with cancer in regard to nutrition
38	The dietitian has a systematic way of documenting nutrition care
39	The treating health service has strong communication and/or shared decision making within the multidisciplinary team about nutrition care
**TRAINING AND EDUCATION**	
40	The treating health service provides training and education support for dietitians about how to deliver and support nutrition care
41	The treating health service ensures nutrition knowledge and skills are maintained and there is little variation between dietitians providing nutrition care
42	The treating health service provides training and education support for medical, nursing, allied health staff and/or those directly involved in nutrition care
43	The treating health service ensures nutrition knowledge and skills are maintained and there is little variation between medical, nursing and/or allied health staff providing and supporting nutrition care
44	The treating health service provides training and education support for dietitians/other staff to support the planning, development, and putting of nutrition care into practice
45	The treating health service provides training and education support for support staff, kitchen staff and/or those indirectly involved in nutrition care

**NUTRITION GOVERNANCE AND DATA**	
46	The treating health service is clear about the roles and responsibilities for nutrition care
47	The treating health service has systems including quality measures and audits of nutrition care practices
48	The treating health service has relevant nutrition policies and guidelines to support nutrition care
49	The treating health service collects data to show the value of nutrition care and any possible cost savings
**PEOPLE WITH CANCER AND CARER INPUT**	
50	People with cancer and their carers have input and feedback into nutrition care and how services work
51	People with cancer and their carers are well aware of what nutrition services exist and the availability of dietitians within their treating health service following a cancer diagnosis
52	People with cancer and their carers are well aware of what community-based nutrition services exist and the availability of dietitians outside of their treating health service following a cancer diagnosis

GENERAL COMMENTS ABOUT CLINICAL DETERMINANTS

Are there any additional items that you consider important as a determinant for (or a factor that influences) the implementation of cancer nutrition care into clinical practice in Australia?

We will be in contact to invite you to participate in Round 2 survey of this study.

Thank you for your time and participation in this survey.

PART B: Round 2 survey

Delphi survey - Round 2

INTRODUCTION AND PLAIN LANGUAGE STATEMENT

STUDY TITLE: Building consensus and identifying priorities: using a Delphi technique to define core elements and clinical determinants for a nutrition model of care for people with cancer
You are invited to participate in the Round 2 survey of this research study examining the core elements and clinical determinants for a nutrition model of care for people with cancer. Our aim is to seek consensus and identify priorities from cancer consumers and cancer nutrition experts using a two-round Delphi technique.

If you cannot complete the survey in one sitting, you can click on the 'Save and Return Later' button to receive a unique return code to retain and use at another time. Alternatively, you can receive your code by contacting us via the details below.

Please download the relevant Plain Language Statement (PLS) here:

PLS cancer consumers.pdf

PLS cancer nutrition experts.pdf

Please direct any questions about this research to Jenelle Loeliger (Jenelle.Loeliger@petermac.org) or Nicole Kiss (nicole.kiss@deakin.edu.au). This study has received ethics approval at Deakin University (reference number: 2024/HE000624).

ELIGIBILITY QUESTIONS

Have you read and understood the Plain Language Statement previously attached?	○ Yes<br>○ No (please direct questions to contact persons noted previously)
Do you meet the eligibility criteria as outlined in the Plain Language Statement and below?<br><br>For cancer consumers:<br><br>Aged ≥18 years Live in Australia Previously diagnosed with cancer or a caregiver for someone who has Able to read and understand English to a proficient level Access to the internet to complete an online survey For cancer nutrition experts:<br><br>One or more of the following: Dietitian with ≥3 years full-time experience (or pro-rata for part-time) managing/treating people with cancer in any healthcare setting; AND/OR Any healthcare clinician or health professional with ≥5 years in any healthcare setting managing/treating people with cancer and working alongside a dietitian within a multidisciplinary team; AND/OR Researcher or academic who has published in the field of cancer and nutrition in the past 5 years. Aged ≥18 years Live and work in Australia Able to read and understand English to a proficient level Access to the internet to complete an online survey	○ Yes<br>○ No (do not proceed any further)
Do you consent to participating and wish to proceed to Round 2 of this Delphi survey?	○ Yes<br>○ No (do not proceed any further)

CONTACT DETAILS AND GROUP

This is to confirm your email address for the purpose of inviting you to participate in a Round 3 survey (if applicable) and which group you best fit with.

What is your email address?

____

Which group below do you belong to?

◯ Cancer consumer - person with cancer or carer of someone with cancer
◯ Cancer nutrition expert - healthcare professional and/or academic

Core elements of a nutrition model of care for people with cancer

Consumers

We are seeking your opinion on what makes up the essential or core elements of a nutrition model of care for people with cancer. A recent scoping review of the published literature identified 10 elements from within various cancer nutrition models of care.

In the Round 1 survey of this project, 6 out of 10 core elements were rated as having strong consensus (i.e., both highly rated for importance and feasibility: Core elements with strong consensus.pdf) and therefore do not appear again in this Round 2 survey. The remaining 4 core elements appear again in this Round 2 survey for your rating (i.e., core elements 5, 7, 8 and 10). In addition, a summary of the overall group rating for each item and your own individual rating (if completed) from the Round 1 survey is provided.

We would like your thoughts on (1) how important each of the proposed elements are to support a cancer nutrition model of care, and (2) how feasible you think the element is to have incorportated or put into care within a health service, based on your experience. You will be asked to rate importance on an 11-point Likert scale with 0 = not at all important, through to 10 = extremely important. Feasibility will be rated similarly on an 11-point Likert scale with 0 = not at all feasible, through to 10 = extremely feasible. An unable to rate option is also available. You can also make comments about the elements proposed and describe any additional elements you consider to be important.

From these survey questions, we are aiming to gain expert opinion from across Australia to come up with a set of core elements that supports best practice nutrition care for people with cancer in Australia.

Cancer nutrition experts

We are seeking your opinion on what makes up the essential or core elements of a nutrition model of care for people with cancer. A recent scoping review of the published literature identified 10 elements that underpinned cancer nutrition models of care.

In the Round 1 survey of this project, 6 out of 10 core elements were rated as having strong consensus (i.e., both highly rated for importance and feasibility: Core elements with strong consensus.pdf) and therefore do not appear again in this Round 2 survey. The remaining 4 core elements appear again in this Round 2 survey for your rating (i.e., core elements 5, 7, 8 and 10). In addition, a summary of the overall group rating for each item and your own individual rating (if completed) from the Round 1 survey is provided.

We would like your thoughts on (1) how important each of the proposed elements are in underpinning a cancer nutrition model of care in clinical practice, and (2) how feasible you think the proposed element is to implement into clinical practice, based on your experience and expertise. You will be asked to rate importance on an 11-point Likert scale with 0 = not at all important, through to 10 = extremely important. Feasibility will be rated similarly on an 11-point Likert scale with 0 = not at all feasible, through to 10 = extremely feasible. An unable to rate option is also available. You can also make comments about the elements proposed and describe any additional elements you consider to be important.

From these survey questions, we are aiming to gain expert opinion from across Australia to define a set of core elements that supports best practice nutrition care for people with cancer in Australia.

CORE ELEMENT 5

Consumers

CORE ELEMENT 5. Nutrition care is easy to access and provided in a timely manner.

Nutrition models of care are developed to support access to expert nutrition care for people with cancer. They are easy, timely, flexible and offer choice in the type of care e.g., face-to-face or telehealth. Models support people with cancer to have access to the right nutrition care at the right time; and broadly raise awareness about the importance of nutrition, where to access evidence-based nutrition information, and the nutrition services available to them.

Additional information/examples: models with quick and easy nutrition screening processes to identify nutrition risk and nutrition information proactively provided to people with cancer; models that incorporate phone or video-telehealth consultations as an option, standard care or as an alternative to face-to-face care for patient and carer convenience.

Round 1 summary:

[redacted]

Cancer nutrition experts

CORE ELEMENT 5. Nutrition care is easy to access and provided in a timely manner.

Nutrition models of care are developed to support access to expert nutrition care for people with cancer. They are easy, proactive, timely, flexible and offer choice in different modes of care e.g., face-to-face or telehealth. Models support people with cancer to have access to the right nutrition care at the right time and broadly raise awareness about the importance of nutrition, where to access evidence-based nutrition information and the nutrition services available to them.

Additional information/examples: models that incorporate the use of blanket/automatic criteria and referrals to the dietitian for high nutrition risk groups; models with clear nutrition screening processes to identify nutrition risk and nutrition information proactively provided to people with cancer; models that incorporate phone or video-telehealth consultations as an option, standard care or as an alternative to face-to-face care for patient and carer convenience.

Round 1 summary:

[redacted]

Please rate on the following scale: 0 = not at all important/feasible, to 10 = extremely important/feasible

	0	1	2	3	4	5	6	7	8	9	10	Unable to rate
How IMPORTANT do you think this element is in underpinning and supporting a nutrition model of care for people with cancer?	○	○	○	○	○	○	○	○	○	○	○	○
How FEASIBLE do you think this element is to incorporate and adopt into a clinical setting/your health service?	○	○	○	○	○	○	○	○	○	○	○	○

Please share any reasons for your answer or other comments about this item (not already raised in Round 1 survey):

What could make this item more feasible (particularly considering solutions within current resources)?

CORE ELEMENT 7

Consumers

CORE ELEMENT 7. Nutrition care has strong multidisciplinary team engagement and leadership.

Strong multidisciplinary teamwork including nutrition expert leadership, is used before, during and after a nutrition model of care goes into clinical practice. Leadership and support are gained from a range of decision makers in the health service when developing and putting a nutrition model of care into place.

Additional information/examples: the multidisciplinary team has input into developing the model and part of decision making around nutrition care; models that are new and reasonable options for care are explored and if they work, they are supported by the wider team.

Round 1 summary:

Cancer nutrition experts

CORE ELEMENT 7. Nutrition care has strong multidisciplinary team engagement and leadership.

Before, during and after implementation of a nutrition model of care, strong multidisciplinary team engagement including nutrition expert leadership, is embedded within the team environment. Clinical, operational, and executive leadership and support for evidence-based nutrition care is gained for the development and implementation of nutrition models of care into clinical practice.

Additional information/examples: the dietitian and nutrition care is embedded into the multidisciplinary team processes such as within team meetings, ward rounds and multimodal programs of care; models should have the support of the multidisciplinary team through input into developing and refining the model and be embedded into clinical decision making around nutrition care; models that are novel, pragmatic, feasible and appropriate to the setting are explored and when demonstrated to be feasible, supported by the multidisciplinary team and key decision makers.

Round 1 summary:

Please rate on the following scale: 0 = not at all important/feasible, to 10 = extremely important/feasible

	0	1	2	3	4	5	6	7	8	9	10	Unable to rate
How IMPORTANT do you think this element is in underpinning and supporting a nutrition model of care for people with cancer?	○	○	○	○	○	○	○	○	○	○	○	○
How FEASIBLE do you think this element is to incorporate and adopt into a clinical setting/your health service?	○	○	○	○	○	○	○	○	○	○	○	○

Please share any reasons for your answer or other comments about this item (not already raised in Round 1 survey):

What could make this item more feasible (particularly considering solutions within current resources)?

CORE ELEMENT 8

Consumers

CORE ELEMENT 8. Nutrition care is supported across all time-points of the cancer care continuum and different settings.

Nutrition models of care support the best nutrition care across all time-points in the cancer care pathway including before, during and after treatment. In addition, models support nutrition care in more than one setting within a single health service (i.e., between inpatients and outpatients) and between different settings and providers (i.e., between hospitals, between private/public providers, between hospitals/primary and community care).

Additional information/examples: models that provide nutrition care before, during and after treatment across both inpatients and outpatients e.g., dietitian sees patient in hospital outpatient clinic before surgery, during a hospital stay, after going home in a hospital outpatient clinic, longer-term either in a hospital outpatient clinic and/or community health; models that support transitions of care between hospital and home e.g., a regional patient is seen by dietitian after surgery as an inpatient at a city hospital and then referred to a dietitian located closer to home in primary/community care for longer-term nutrition care.

Round 1 summary:

Cancer nutrition experts

CORE ELEMENT 8. Nutrition care is supported across all time-points of the cancer care continuum and different settings.

Nutrition models of care embed patient-centred and appropriate nutrition care across all time-points in the cancer care continuum (before, during and after treatment), adapted to the individual and the setting. In addition, models support nutrition care in more than one setting within a single health service (i.e., between inpatients and outpatients) and between different settings, providers and sectors (i.e., between hospitals, between private/public providers, between acute/primary and community care).

Additional information/examples: models that provide nutrition care before, during and after treatment into survivorship across both inpatients and outpatients e.g., dietitian sees patient in hospital outpatient clinic before stem cell transplant, during their inpatient admission, in hospital outpatient clinic after discharge, longer-term as needed in hospital outpatient clinic and/or community/primary care; models that support transitions of care between hospital and home e.g., a regional patient is seen by dietitian after surgery as an inpatient and in outpatient clinic during radiotherapy at a metropolitan hospital and then referred to a dietitian located closer to home in community/primary care for further post-treatment nutrition care or a shared-care arrangement between metropolitan and regional providers.

Round 1 summary:

Please rate on the following scale: 0 = not at all important/feasible, to 10 = extremely important/feasible

	0	1	2	3	4	5	6	7	8	9	10	Unable to rate
How IMPORTANT do you think this element is in underpinning and supporting a nutrition model of care for people with cancer?	○	○	○	○	○	○	○	○	○	○	○	○
How FEASIBLE do you think this element is to incorporate and adopt into a clinical setting/your health service?	○	○	○	○	○	○	○	○	○	○	○	○

Please share any reasons for your answer or other comments about this item (not already raised in Round 1 survey):

What could make this item more feasible (particularly considering solutions within current resources)?

CORE ELEMENT 10

Consumers

CORE ELEMENT 10. Nutrition care is supported by data and data is integrated into usual processes.

Models of nutrition care are supported by and use data appropriate to the setting. Data is used from the electronic medical record (EMR) or other data systems and from audits to improve information sharing across multidisciplinary teams and people with cancer/their carers.

Additional information/examples: models can include sharing of key information between a dietitian and patient/carer before or after a consultation; models can be monitored through audits or data that helps guide improvements to care.

Round 1 summary:

[redacted]

Cancer nutrition experts

CORE ELEMENT 10. Nutrition care is supported by data and data is integrated into usual processes.

Models of nutrition care are supported by and utilise data appropriate to the setting. Data integration via an electronic medical record (EMR) or alternative robust data system are utilised to improve real-time operational workflows, incorporate audit and feedback within a quality improvement system and to aid information sharing across multidisciplinary teams and with people with cancer and their carers.

Additional information/examples: models can be supported by an EMR to generate automatic referrals to the dietitian, data fields for key patient-reported and clinical outcomes i.e., risk status, malnutrition diagnosis; models can include sharing of meaningful data and information with patients/carers before or after a dietitian consultation; models can incorporate dashboards/databases/nutrition data reported in a multidisciplinary meeting from an EMR/other data system to highlight nutrition risk and facilitate nutrition interventions; models can be monitored in real-time through the use of key performance indicators or audits that guide targeted care improvements.

Round 1 summary:

[redacted]

Please rate on the following scale: 0 = not at all important/feasible, to 10 = extremely important/feasible

	0	1	2	3	4	5	6	7	8	9	10	Unable to rate
How IMPORTANT do you think this element is in underpinning and supporting a nutrition model of care for people with cancer?	○	○	○	○	○	○	○	○	○	○	○	○
How FEASIBLE do you think this element is to incorporate and adopt into a clinical setting/your health service?	○	○	○	○	○	○	○	○	○	○	○	○

Please share any reasons for your answer or other comments about this item (not already raised in Round 1 survey):

What could make this item more feasible (particularly considering solutions within current resources)?

GENERAL COMMENTS ABOUT CORE ELEMENTS
Are there any additional core/essential elements you consider important to underpin and support a cancer nutrition model of care?

	Key clinical determinants of cancer nutrition care
**Consumers**	We are seeking your opinion about the key clinical determinants for (or factors that influence and/or concerns that need to be considered), the successful implementation of cancer nutrition care into clinical practice.<br><br>The following list of items have been made by the research team. In the Round 1 survey of this project, 17 out of 52 clinical determinants were rated as having strong consensus (i.e., both highly rated for importance and feasibility: Clinical determinant with strong consensus.pdf) and therefore do not appear again in this Round 2 survey. The remaining 35 clinical determinants appear again in this Round 2 survey for your rating (numbered as per Round 1). In addition, a summary of the overall group rating and your individual rating (if completed) for each item from the Round 1 survey is provided.<br><br>We would like your thoughts on how important each of the proposed items are as a determinant for (or a factor that influences) the implementation of cancer nutrition care into clinical care in Australia. We would also like your thoughts on how feasible you think the item is to put into place into clinical care, based on your experience. You will be asked to rate importance on a 11-point Likert scale with 0 = not at all important, through to 10 = extremely important. Feasibility will be rated similarly on a 11-point Likert scale with 0 = not at all feasible, through to 10 = extremely feasible. An unable to rate option is also available. You can provide reasons or comments for each item and describe any additional items you consider to be important.<br><br>This work is aiming to work out the most important factors and/or concerns that need to be thought about and addressed to support the implementation of the best possible models of nutrition care for people with cancer Australia-wide.
**Cancer nutrition experts**	We are seeking your opinion about the key clinical determinants for (or factors that influence and/or concerns that need to be considered), the successful implementation of cancer nutrition care into clinical practice.<br><br>The following list of proposed items have been generated by the research team. In the Round 1 survey of this project, 17 out of 52 clinical determinants were rated as having strong consensus (i.e., both highly rated for importance and feasibility: Clinical determinant with strong consensus.pdf) and therefore do not appear again in this Round 2 survey. The remaining 35 clinical determinants appear again in this Round 2 survey for your rating (numbered as per Round 1). In addition, a summary of the overall group rating and your individual rating (if completed) for each item from the Round 1 survey is provided.<br><br>We would like your thoughts on how important each of the proposed items are as a determinant for (or a factor that influences) the implementation of cancer nutrition care into clinical practice in Australia. We would also like your thoughts on how feasible you think the proposed item is to implement into clinical practice, based on your experience and expertise. You will be asked to rate importance on a 11-point Likert scale with 0 = not at all important, through to 10 = extremely important. Feasibility will be rated similarly on a 11-point Likert scale with 0 = not at all feasible, through to 10 = extremely feasible. An unable to rate option is also available. You can provide reasons or comments for each item and describe any additional items you consider to be important.<br><br>This work is aiming to identify and prioritise the most important factors and/or concerns that need to be considered and addressed to support the implementation of optimal and equitable models of nutrition care for people with cancer across healthcare settings in Australia.

Scale and questions asked against each proposed item:

Please rate on the following scale: 0 = not at all important/feasible, to 10 = extremely important/feasible.

	0	1	2	3	4	5	6	7	8	9	10	Unable to rate
How IMPORTANT do you think this proposed item is as a determinant for (or a factor that influences) the implementation of cancer nutrition care into clinical practice in Australia?	○	○	○	○	○	○	○	○	○	○	○	○
How FEASIBLE do you think this proposed item is to implement into clinical practice/your health service?	○	○	○	○	○	○	○	○	○	○	○	○

Please share any reasons for your answer or other comments about this item (not already raised in Round 1 survey):

What could make this item more feasible (particularly considering solutions within current resources)?

Proposed items (with a Round 1 summary provided against each item – both group rating and participants individual rating)

2	The treating health service has dedicated resourcing/funding, support and systems for *outpatient* nutrition clinics and care
4	The treating health service has a stable dietitian workforce with manageable levels of staff turnover
5	The treating health service has senior and/or specialist oncology dietitians
6	The treating health service supports the dietitian to have enough time to provide nutrition care to patients (both inpatients and outpatients) and supports the development and monitoring of care models
7	In primary and community care, there are resources for and availability of dietitians providing nutrition care to people with cancer
8	Primary and community care has dietitians who have experience in providing cancer nutrition care to people with cancer
**ENVIRONMENT, SPACE AND TECHNOLOGY**	
9	The treating health service has a physical space for the dietitian to see patients in the outpatient environment

[redacted]	
11	The treating health service has clinic space for the dietitian with the multidisciplinary team in the same area/location (i.e., co-located)
[redacted]	
**STAKEHOLDER ENGAGMENT, AWARENESS AND SUPPORT**	
13	The nutrition service has buy-in and support from key decision makers in the health service
[redacted]	
**ACESS TO CARE AND CLINICAL PRACTICES**	
15	Patients and their carers have access to free or low-cost nutrition services i.e., part of standard cancer care
[redacted]	
17	The treating health service provides patients and their carers access to a dietitian and support for nutrition care *between diagnosis and starting treatment* (i.e., pre-surgery, pre-chemotherapy, pre-radiotherapy)
[redacted]	
19	The treating health service provides patients and their carers access to a dietitian and support for nutrition care *up to 3 months after treatment*
20	The treating health service provides patients and their carers access to a dietitian and support for nutrition care *>3 months post treatment* (i.e., long-term survivorship including living with advanced cancer)
21	The treating health service provides patients and their carers access and directions for nutrition care between health providers

	and/or settings (i.e., from one hospital to another, public to private provider or vice versa, from hospital to primary or community care provider)
22	Patients and their carers have access to a dietitian and support for nutrition care in primary and community care as needed
[redacted]	
24	Patients and their carers have access to a dietitian and nutrition care when considered at *moderate* risk of malnutrition and/or poor nutrition
25	Patients and their carers have access to a dietitian and nutrition care when considered at *low* risk of malnutrition and/or poor nutrition
[redacted]	
27	The treating health service has a system for the early identification of nutrition needs for people after a cancer diagnosis
[redacted]	
32	There is a process for patients and their carers who are not able to be seen by a dietitian to receive nutrition information and care i.e., referral to community services, directed to online resources or programs
33	The treating health service uses a system where nutrition care for patients with malnutrition and sarcopenia (or other nutrition

	disorders) will be seen before patients with issues that could lead to long term poor health e.g., weight (fat) gain, heart issues
[redacted]	
37	The dietitian monitors people with cancer in regard to nutrition
[redacted]	
39	The treating health service has strong communication and/or shared decision making within the multidisciplinary team about nutrition care
**TRAINING AND EDUCATION**	
40	The treating health service provides training and education support for dietitians about how to deliver and support nutrition care
41	The treating health service ensures nutrition knowledge and skills are maintained and there is little variation between dietitians providing nutrition care
42	The treating health service provides training and education support for medical, nursing, allied health staff and/or those directly involved in nutrition care
43	The treating health service ensures nutrition knowledge and skills are maintained and there is little variation between medical, nursing and/or allied health staff providing and supporting nutrition care
44	The treating health service provides training and education support for dietitians/other staff to support the planning, development, and putting of nutrition care into practice
45	The treating health service provides training and education support for support staff, kitchen staff and/or those indirectly involved in nutrition care

**NUTRITION GOVERNANCE AND DATA**	
46	The treating health service is clear about the roles and responsibilities for nutrition care
47	The treating health service has systems including quality measures and audits of nutrition care practices
49	The treating health service collects data to show the value of nutrition care and any possible cost savings
**PEOPLE WITH CANCER AND CARER INPUT**	
50	People with cancer and their carers have input and feedback into nutrition care and how services work
51	People with cancer and their carers are well aware of what nutrition services exist and the availability of dietitians within their treating health service following a cancer diagnosis
52	People with cancer and their carers are well aware of what community-based nutrition services exist and the availability of dietitians outside of their treating health service following a cancer diagnosis

GENERAL COMMENTS ABOUT CLINICAL DETERMINANTS

Are there any additional items that you consider important as a determinant for (or a factor that influences) the implementation of cancer nutrition care into clinical practice in Australia?

Are you happy to be contacted by the researchers about future studies being conducted on this topic?

◯ Yes
◯ No

Thank you for your time and participation in this survey.
