# Supplementary material for: Building consensus and identifying priorities: using a Delphi technique to define core elements and clinical determinates for a nutrition model of care for people with cancer
Source: J Cancer Surviv. 2026 Feb 13;20(Suppl 2):1543–52. doi: 10.1007/s11764-026-01984-9 (PMC13744544; doi:10.1007/s11764-026-01984-9)
Supplement: Supplementary file 2 [file 11764_2026_1984_MOESM2_ESM.pdf]

**Supplementary Material 2 (ESM_2)**

# Building consensus and identifying priorities: using a Delphi technique to define core elements and clinical determinates for a nutrition model of care for people with cancer

J. Loeliger [a, b, c, d], A. Ugalde [e], J. Porter [b, c], N. Kiss [b, c]

[a] Nutrition and Speech Pathology Department, Peter MacCallum Cancer Centre, ,305 Grattan Street Melbourne VIC 3000, Australia

[b] Institute for Physical Activity and Nutrition (IPAN), Deakin University, 1 Gheringhap Street Geelong VIC 3220, Australia

[c] School of Exercise and Nutrition Sciences, Deakin University, 1 Gheringhap Street Geelong VIC 3220, Australia

[d] Sir Peter MacCallum Department of Oncology, University of Melbourne, Grattan Street, Parkville VIC 3010, Australia

[e] Institute for Health Transformation, Quality and Patient Safety, Deakin University, 1 Gheringhap Street Geelong VIC 3220, Australia

**Corresponding author**:

Jenelle Loeliger

Email: jenelle.loeliger@petermac.org

Phone: +613 8559 5161

## Contents

Table A. Summary of results for *core elements* from Round 1 and 2 Delphi surveys ........ 3

Table B. Summary of results for *clinical determinants* from Round 1 and 2 Delphi surveys ........ 5

# Table A. Summary of results for *core elements* from Round 1 and 2 Delphi surveys

| Proposed item | | Round 1 (R1) | | | | | | | | | | | Round 2 (R2) | | | | | | | | | | |
|---|---|---|---|---|---|---|---|---|---|---|---|---|---|---|---|---|---|---|---|---|---|---|---|
| | | Importance | | | | | Feasibility | | | | | R1 combined cons. rating[c] | Importance | | | | | Feasibility | | | | | R2 combined cons. rating[c] |
| | | Median (IQR)[a] | % rated[b] | | | Cons. rating[c] | Median (IQR)[a] | % rated[b] | | | Cons. rating[c] | | Median (IQR)[a] | % rated[b] | | | Cons. rating[c] | Median (IQR)[a] | % rated[b] | | | Cons. rating[c] | |
| | | | L | M | H | | | L | M | H | | | | L | M | H | | | L | M | H | | |
| 1 | Nutrition care is timely, evidence-based and clearly documented via use of a care pathway, protocol and/or structured clinic | 10.0 (9.0-10.0) | 1.4 | 2.9 | 95.7 | S | 8.0 (7.0-9.0) | 2.9 | 17.4 | 79.7 | S | **S** | N/A | | | | | | | | | | |
| 2 | Nutrition care incorporates nutrition expertise, leadership and decision making by a dietitian or other health professional | 10.0 (9.0-10.0) | 0.0 | 2.9 | 97.1 | S | 8.0 (6.0-10.0) | 5.8 | 23.2 | 71.0 | S | **S** | N/A | | | | | | | | | | |
| 3 | Nutrition care is based on evidence but is also flexible and integrated into existing processes and local environments | 10.0 (9.0-10.0) | 0.0 | 1.4 | 98.6 | S | 8.0 (7.0-10.0) | 4.3 | 14.5 | 81.2 | S | **S** | N/A | | | | | | | | | | |
| 4 | Nutrition care includes multi-directional communication between the person with cancer, their carer and health professionals | 10.0 (9.0-10.0) | 0.0 | 4.3 | 95.7 | S | 8.0 (6.0-10.0) | 5.8 | 20.3 | 72.5 | S | **S** | N/A | | | | | | | | | | |
| 5 | Nutrition care is easy to access and provided in a timely manner | 10.0 (9.0-10.0) | 1.4 | 1.4 | 97.1 | S | 7.0 (6.0-9.0) | 5.8 | 29.0 | 65.2 | M | **M** | 10.0 (9.0-10.0) | 0.0 | 0.0 | 98.3 | S | 8.0 (6.0-8.0) | 3.3 | 23.3 | 73.3 | S | **S** |
| 6 | Nutrition care includes risk stratification | 10.0 (8.0-10.0) | 2.9 | 5.8 | 91.3 | S | 8.0 (6.0-9.0) | 7.2 | 21.7 | 71.0 | S | **S** | N/A | | | | | | | | | | |
| 7 | Nutrition care has multidisciplinary team engagement and leadership | 10.0 (9.0-10.0) | 1.4 | 4.3 | 94.2 | S | 7.0 (6.0-9.0) | 7.2 | 26.1 | 66.7 | M | **M** | 10.0 (9.0-10.0) | 0.0 | 3.3 | 96.7 | S | 7.5 (6.8-9.0) | 6.7 | 18.3 | 75.0 | S | **S** |
| 8 | Nutrition care is supported across all time-points of the cancer care | 10.0 (9.0-10.0) | 1.4 | 2.9 | 95.7 | S | 7.0 (5.0-8.0) | 15.9 | 31.9 | 52.2 | M | **M** | 10.0 (9.0-10.0) | 1.7 | 1.7 | 96.7 | S | 7.0 (6.0-8.0) | 10.0 | 28.3 | 61.7 | M | **M** |

| | | | | | | | | | | | | | | | | | | | | | | | |
|---|---|---|---|---|---|---|---|---|---|---|---|---|---|---|---|---|---|---|---|---|---|---|---|
| | continuum and different settings | | | | | | | | | | | | | | | | | | | | | | |
| 9 | Nutrition care is supported by training and education | 10.0 (9.0-10.0) | 1.4 | 5.8 | 92.8 | S | 8.0 (6.0-9.0) | 8.7 | 17.4 | 72.5 | S | **S** | N/A | | | | | | | | | | |
| 10 | Nutrition care is supported by data and data is integrated into usual processes | 10.0 (8.0-10.0) | 0.0 | 1.4 | 95.7 | S | 6.5 (5.0-8.0) | 10.1 | 39.1 | 49.3 | W | **M** | 9.0 (8.0-10.0) | 0.0 | 1.7 | 98.3 | S | 7.0 (6.0-8.0) | 8.3 | 26.7 | 63.3 | M | **M** |

[a]Ratings were on an 11-point unipolar Likert scale from '0' (not at all important/feasible) to '10' (extremely important/feasible), or unable to rate; [b]rating low = Likert score <4: rating moderate = Likert score 4-<7: rating high = Likert score ≥7-10: unable to rate or missing data not reported under any rating; [c]*S* = Strong consensus i.e., ≥70% of participants indicated a highly rated response for both important and feasibility (cell coloured green): *M* = Medium consensus i.e., ≥70% of participants indicated a highly rated response for one of importance or feasibility and between 50% and <70% indicated a highly rated response for the alternate of either importance or feasibility (cell coloured yellow): *W* = Weak consensus i.e., <50% of participants indicated a highly rated response for both importance and feasibility or one <50% and the alternate <70% (cell coloured orange).

Abbreviations: R1=Round1; R2=Round 2; IQR=Interquartile range; L=low; M=moderate; H=high; Consensus=cons.

# Table B. Summary of results for *clinical determinants* from Round 1 and 2 Delphi surveys

| Category/Proposed item | | Round 1 (R1) | | | | | | | | | | | Round 2 (R2) | | | | | | | | | | |
|---|---|---|---|---|---|---|---|---|---|---|---|---|---|---|---|---|---|---|---|---|---|---|---|
| | | Importance | | | | | Feasibility | | | | | R1 combined cons. rating[c] | Importance | | | | | Feasibility | | | | | R2 combined cons. rating[c] |
| | | Median (IQR)[a] | % rated[b] L | % rated[b] M | % rated[b] H | Cons. rating[c] | Median (IQR)[a] | % rated[b] L | % rated[b] M | % rated[b] H | Cons. rating[c] | | Median (IQR)[a] | % rated[b] L | % rated[b] M | % rated[b] H | Cons. rating[c] | Median (IQR)[a] | % rated[b] L | % rated[b] M | % rated[b] H | Cons. rating[c] | |
| STAFFING AND RESOURCES | | | | | | | | | | | | | | | | | | | | | | | |
| 1 | The treating health service has dedicated resourcing/funding and availability of dietitians working in oncology | 10.0 (10.0-10.0) | 0.0 | 2.9 | 97.1 | S | 7.0 (5.0-8.0) | 11.6 | 36.2 | 50.7 | M | **M** | 10.0 (9.0-10.0) | 0.0 | 3.3 | 96.7 | S | 7.0 (5.0-8.0) | 11.7 | 28.3 | 60.0 | M | **M** |
| 2 | The treating health service has dedicated resourcing/funding, support and systems for outpatient nutrition clinics and care | 10.0 (9.0-10.0) | 1.4 | 2.9 | 95.7 | S | 6.0 (5.0-8.0) | 14.5 | 39.1 | 44.9 | W | **M** | 10.0 (9.0-10.0) | 0.0 | 0.0 | 100.0 | S | 7.0 (5.0-8.0) | 13.3 | 28.3 | 58.3 | M | **M** |
| 3 | The treating health service has dedicated resourcing/funding, support and systems for inpatient nutrition care | 10.0 (9.0-10.0) | 0.0 | 2.9 | 97.1 | S | 8.0 (7.0-10.0) | 4.3 | 18.8 | 72.5 | S | **S** | N/A | | | | | | | | | | |
| 4 | The treating health service has a stable dietitian workforce with manageable levels of staff turnover | 9.0 (8.0-10.0) | 2.9 | 10.1 | 87.0 | S | 6.0 (5.0-8.0) | 13.0 | 37.7 | 46.4 | W | **M** | 9.0 (8.0-10.0) | 0.0 | 5.0 | 95.0 | S | 6.0 (5.0-8.0) | 8.3 | 43.3 | 48.3 | W | **M** |
| 5 | The treating health service has senior and/or specialist oncology dietitians | 10.0 (8.0-10.0) | 1.4 | 4.3 | 94.2 | S | 7.0 (6.0-9.0) | 13.0 | 23.2 | 60.9 | M | **M** | 10.0 (9.0-10.0) | 1.7 | 3.3 | 95.0 | S | 7.5 (6.0-9.0) | 10.0 | 20.0 | 70.0 | S | **S** |
| 6 | The treating health service supports the dietitian to have adequate time to provide nutrition care to patients (both inpatients and outpatients) and supports the development and monitoring of appropriate care models | 10.0 (9.0-10.0) | 1.4 | 2.9 | 95.7 | S | 7.0 (5.0-8.0) | 14.5 | 33.3 | 49.3 | W | **M** | 10.0 (9.8-10.0) | 0.0 | 1.7 | 98.3 | S | 7.0 (5.8-8.0) | 15.0 | 18.3 | 66.7 | M | **M** |
| 7 | In primary and community care, there are resources | 10.0 (9.0-10.0) | 0.0 | 4.3 | 95.7 | S | 7.0 (5.0-8.0) | 13.0 | 31.9 | 52.2 | M | **M** | 10.0 (9.0-10.0) | 0.0 | 3.3 | 95.0 | S | 7.0 (5.0-8.0) | 10.0 | 35.0 | 53.3 | M | **M** |

| | | | | | | | | | | | | | | | | | | | | | | | |
|---|---|---|---|---|---|---|---|---|---|---|---|---|---|---|---|---|---|---|---|---|---|---|---|
| | for and availability of dietitians providing nutrition care to people with cancer | | | | | | | | | | | | | | | | | | | | | | |
| 8 | Primary and community care has dietitians who have experience in providing nutrition care to people with cancer | 10.0 (8.0-10.0) | 0.0 | 7.2 | 89.9 | S | 6.0 (5.0-8.0) | 10.1 | 47.8 | 37.7 | W | M | 10.0 (8.0-10.0) | 0.0 | 5.0 | 93.3 | S | 6.0 (5.0-7.0) | 6.7 | 46.7 | 43.3 | W | M |
| ENVIRONMENT, SPACE AND TECHNOLOGY | | | | | | | | | | | | | | | | | | | | | | | |
| 9 | The treating health service has a physical space for the dietitian to see patients in the outpatient environment | 10.0 (8.0-10.0) | 4.3 | 10.1 | 85.5 | S | 7.0 (4.5-9.0) | 17.4 | 27.5 | 52.2 | M | M | 10.0 (8.5-10.0) | 1.7 | 10.0 | 86.7 | S | 7.0 (5.0-9.0) | 10.0 | 33.3 | 56.7 | M | M |
| 10 | The treating health service has access to phone and video-telehealth technology for dietitian consultations | 10.0 (9.0-10.0) | 0.0 | 7.2 | 91.3 | S | 9.0 (7.8-10.0) | 4.3 | 13.0 | 81.2 | S | S | N/A | | | | | | | | | | |
| 11 | The treating health service has clinic space for the dietitian with the multidisciplinary team in the same area/location (i.e., co-located) | 9.0 (8.0-10.0) | 1.4 | 5.8 | 92.8 | S | 6.0 (5.0-7.0) | 13.0 | 49.3 | 34.8 | W | M | 9.0 (8.0-10.0) | 1.7 | 6.7 | 91.7 | S | 6.0 (5.0-8.0) | 13.3 | 45.0 | 41.7 | W | M |
| 12 | The nutrition service has access to tools and equipment to support evidence-based nutrition screening, assessment, diagnosis, and treatment of nutrition disorders | 10.0 (9.0-10.0) | 0.0 | 2.9 | 97.1 | S | 8.0 (6.0-9.5) | 2.9 | 23.2 | 71.0 | S | S | N/A | | | | | | | | | | |
| STAKEHOLDER ENGAGEMENT, AWARENESS AND SUPPORT | | | | | | | | | | | | | | | | | | | | | | | |
| 13 | The nutrition service has buy-in and engagement from key operational and clinical stakeholders i.e., managers, directors, executive, multidisciplinary clinicians, and leaders | 10.0 (9.0-10.0) | 0.0 | 2.9 | 97.1 | S | 7.0 (5.5-9.0) | 10.1 | 30.4 | 56.5 | M | M | 10.0 (9.0-10.0) | 0.0 | 0.0 | 98.3 | S | 7.0 (6.0-8.0) | 11.7 | 20.0 | 68.3 | M | M |

| | | | | | | | | | | | | | | | | | | | | | | | |
|---|---|---|---|---|---|---|---|---|---|---|---|---|---|---|---|---|---|---|---|---|---|---|---|
| 14 | The dietitian has clinical support from within the oncology multidisciplinary team e.g., doctors, nurses and others | 10.0 (10.0-10.0) | 0.0 | 1.4 | 98.6 | S | 8.0 (7.0-10.0) | 5.8 | 14.5 | 76.8 | S | **S** | N/A | | | | | | | | | | |
| ACCESS TO CARE AND CLINICAL PRACTICES | | | | | | | | | | | | | | | | | | | | | | | |
| 15 | Patients and their carers have access to free or low-cost nutrition services i.e., part of standard cancer care | 10.0 (9.0-10.0) | 0.0 | 2.9 | 97.1 | S | 8.0 (6.0-9.0) | 5.8 | 29.0 | 63.8 | M | **M** | 10.0 (10.0-10.0) | 0.0 | 0.0 | 100.0 | S | 8.0 (6.0-9.0) | 8.3 | 23.3 | 68.3 | M | **M** |
| 16 | Patients and their carers have access to universal nutrition information or resources i.e., nutrition information and/or interventions that are suitable for all people with cancer e.g., group sessions, written information, a website, app or videos. | 10.0 (9.0-10.0) | 0.0 | 5.8 | 94.2 | S | 8.0 (7.0-10.0) | 5.8 | 15.9 | 76.8 | S | **S** | N/A | | | | | | | | | | |
| 17 | The treating health service provides patients and their carers access to a dietitian and support for nutrition care between diagnosis and starting treatment (i.e., pre-surgery, pre-chemotherapy, pre-radiotherapy or also known as prehabilitation) | 9.0 (8.8-10.0) | 0.0 | 4.3 | 94.2 | S | 6.5 (5.0-8.0) | 13.0 | 34.8 | 47.8 | W | **M** | 9.0 (9.0-10.0) | 3.3 | 1.7 | 93.3 | S | 7.0 (6.0-8.0) | 8.3 | 33.3 | 58.3 | M | **M** |
| 18 | The treating health service provides patients and their carers access to a dietitian and support for nutrition care during treatment | 10.0 (9.0-10.0) | 1.4 | 0.0 | 97.1 | S | 8.0 (7.0-10.0) | 5.8 | 17.4 | 73.9 | S | **S** | N/A | | | | | | | | | | |
| 19 | The treating health service provides patients and their carers access to a dietitian and support for nutrition care up to 3 months after treatment | 10.0 (8.0-10.0) | 0.0 | 8.7 | 91.3 | S | 6.0 (5.0-8.0) | 10.1 | 40.6 | 46.4 | W | **M** | 10.0 (8.0-10.0) | 0.0 | 1.7 | 98.3 | S | 7.0 (5.0-8.0) | 13.3 | 30.0 | 56.7 | M | **M** |

| | | | | | | | | | | | | | | | | | | | | | | | |
|---|---|---|---|---|---|---|---|---|---|---|---|---|---|---|---|---|---|---|---|---|---|---|---|
| 20 | The treating health service provides patients and their carers access to a dietitian and support for nutrition care >3 months post treatment (i.e., long-term survivorship including living with advanced cancer) | 8.0 (7.0-10.0) | 0.0 | 20.3 | 78.3 | S | 6.0 (4.0-7.0) | 18.8 | 42.0 | 33.3 | W | **M** | 8.0 (8.0-10.0) | 0.0 | 13.3 | 86.7 | S | 6.0 (4.0-7.0) | 20.0 | 45.0 | 35.0 | W | **M** |
| 21 | The treating health service provides patients and their carers access to and navigation of nutrition care between health providers and/or settings (i.e., from one hospital to another, public to private provider or vice versa, from hospital to primary or community care provider) | 9.0 (8.0-10.0) | 0.0 | 7.2 | 89.9 | S | 7.0 (5.0-8.0) | 17.4 | 26.1 | 50.7 | M | **M** | 10.0 (8.0-10.0) | 1.7 | 6.7 | 91.7 | S | 7.0 (5.0-8.0) | 15.0 | 26.7 | 58.3 | M | **M** |
| 22 | Patients and their carers have access to a dietitian and support for nutrition care in primary and community care as needed | 9.0 (8.0-10.0) | 5.8 | 5.8 | 87.0 | S | 6.0 (5.0-7.8) | 17.4 | 31.9 | 46.4 | W | **M** | 10.0 (8.0-10.0) | 1.7 | 6.7 | 91.7 | S | 7.0 (5.0-8.0) | 15.0 | 28.3 | 56.7 | M | **M** |
| 23 | Patients and their carers have access to a dietitian and nutrition care when considered at high risk of malnutrition and/or negative nutrition outcomes | 10.0 (10.0-10.0) | 1.4 | 0.0 | 97.1 | S | 9.0 (7.0-10.0) | 5.8 | 14.5 | 75.4 | S | **S** | N/A | | | | | | | | | | |
| 24 | Patients and their carers have access to a dietitian and nutrition care when considered at moderate risk of malnutrition and/or negative nutrition outcomes | 9.0 (8.0-10.0) | 2.9 | 1.4 | 94.2 | S | 7.0 (6.0-9.0) | 5.8 | 24.6 | 63.8 | M | **M** | 10.0 (8.8-10.0) | 1.7 | 3.3 | 95.0 | S | 7.5 (6.0-8.3) | 6.7 | 21.7 | 71.7 | S | **S** |
| 25 | Patients and their carers have access to a dietitian and nutrition care when considered at low risk of malnutrition and/or | 7.0 (5.0-8.0) | 10.1 | 31.9 | 56.5 | M | 5.0 (3.0-6.0) | 29.0 | 43.5 | 21.7 | W | **W** | 7.0 (5.0-9.0) | 6.7 | 31.7 | 61.7 | M | 5.0 (3.0-6.0) | 31.7 | 46.7 | 21.7 | W | **W** |

| | | | | | | | | | | | | | | | | | | | | | | | | |
|---|---|---|---|---|---|---|---|---|---|---|---|---|---|---|---|---|---|---|---|---|---|---|---|---|
| | negative nutrition outcomes | | | | | | | | | | | | | | | | | | | | | | | |
| 26 | The treating health service uses a clearly documented clinical pathway, protocol and/or structured clinic to guide clinical practice and consistent nutrition care/nutrition support | 10.0 (8.0-10.0) | 1.4 | 4.3 | 92.8 | S | 7.5 (6.3-9.0) | 4.3 | 20.3 | 71.0 | S | **S** | N/A | | | | | | | | | | | |
| 27 | The treating health service has a system for the early identification of nutrition needs for people following a cancer diagnosis | 10.0 (9.0-10.0) | 0.0 | 4.3 | 94.2 | S | 8.0 (6.0-9.0) | 8.7 | 18.8 | 68.1 | M | **M** | 10.0 (9.0-10.0) | 0.0 | 1.7 | 98.3 | S | 8.0 (6.0-9.0) | 11.7 | 15.0 | 73.3 | S | **S** |
| 28 | The treating health service has a nutrition screening process including the use of a valid and reliable screening tool (completed following a cancer diagnosis and repeated over time) | 10.0 (9.0-10.0) | 1.4 | 4.3 | 92.8 | S | 8.0 (7.0-10.0) | 5.8 | 15.9 | 73.9 | S | **S** | N/A | | | | | | | | | | | |
| 29 | The treating health service has a process for referral to the dietitian when patient nutrition needs or risks are identified | 10.0 (10.0-10.0) | 0.0 | 1.4 | 97.1 | S | 9.0 (7.5-10.0) | 5.8 | 7.2 | 84.1 | S | **S** | N/A | | | | | | | | | | | |
| 30 | The treating health service uses nutrition risk stratification to support high-risk patients to bypass screening and be seen by the dietitian (i.e., automatic referral based on diagnosis or treatment) e.g., head and neck cancer patients undergoing radiotherapy | 10.0 (10.0-10.0) | 0.0 | 0.0 | 98.6 | S | 9.0 (7.0-10.0) | 2.9 | 13.0 | 78.3 | S | **S** | N/A | | | | | | | | | | | |
| 31 | The treating health service uses nutrition risk stratification, triaging or similar process/method, to support dietitian prioritisation and allocation i.e., to | 10.0 (9.0-10.0) | 1.4 | 2.9 | 94.2 | S | 9.0 (7.0-10.0) | 5.8 | 14.5 | 75.4 | S | **S** | N/A | | | | | | | | | | | |

| | | | | | | | | | | | | | | | | | | | | | | | |
|---|---|---|---|---|---|---|---|---|---|---|---|---|---|---|---|---|---|---|---|---|---|---|---|
| | manage demand/dietitian referrals with limited resources | | | | | | | | | | | | | | | | | | | | | | |
| 32 | If the treating health service uses nutrition risk stratification, triaging or similar process/method, there is a process for patients and their carers who do not meet criteria to be seen by a dietitian or to receive nutrition information and care i.e., referral to community services, directed to online resources or programs | 10.0 (9.0-10.0) | 0.0 | 2.9 | 95.7 | S | 8.0 (5.3-10.0) | 2.9 | 29.0 | 63.8 | M | **M** | 10.0 (9.0-10.0) | 1.7 | 3.3 | 93.3 | S | 7.0 (6.0-9.0) | 10.0 | 20.0 | 68.3 | M | **M** |
| 33 | The treating health service uses a system that prioritises nutrition care for patients with malnutrition and sarcopenia (or other nutrition disorders) over patients with treatment induced issues that could lead to long term poor health e.g., weight (fat mass) gain, cardiotoxicity | 8.0 (7.0-10.0) | 7.2 | 14.5 | 76.8 | S | 8.0 (5.0-8.0) | 7.2 | 29.0 | 55.1 | M | **M** | 8.5 (8.0-10.0) | 8.3 | 6.7 | 81.7 | S | 8.0 (6.0-8.0) | 11.7 | 23.3 | 63.3 | M | **M** |
| 34 | The dietitian has a clear nutrition assessment process including use of a valid and reliable assessment tool (i.e., patient-generated subjective global assessment or subjective global assessment) | 10.0 (8.5-10.0) | 2.9 | 4.3 | 89.9 | S | 9.0 (7.0-10.0) | 5.8 | 10.1 | 79.7 | S | **S** | N/A | | | | | | | | | | |
| 35 | The dietitian provides evidence-based nutrition interventions to patients (i.e., aligned to evidence-based guidelines) | 10.0 (10.0-10.0) | 2.9 | 2.9 | 92.8 | S | 9.0 (7.5-10.0) | 4.3 | 10.1 | 82.6 | S | **S** | N/A | | | | | | | | | | |

| | | | | | | | | | | | | | | | | | | | | | | | |
|---|---|---|---|---|---|---|---|---|---|---|---|---|---|---|---|---|---|---|---|---|---|---|---|
| 36 | The dietitian diagnoses relevant conditions e.g., malnutrition, sarcopenia | 10.0 (10.0-10.0) | 4.3 | 1.4 | 92.8 | S | 9.0 (7.5-10.0) | 5.8 | 8.7 | 82.6 | S | **S** | N/A | | | | | | | | | | |
| 37 | The dietitian provides nutrition monitoring for people with cancer and has a process of nutrition evaluation | 10.0 (9.0-10.0) | 0.0 | 8.7 | 87.0 | S | 8.0 (6.0-10.0) | 7.2 | 18.8 | 66.7 | M | **M** | 10.0 (9.0-10.0) | 0.0 | 5.0 | 95.0 | S | 8.0 (6.8-9.0) | 6.7 | 18.3 | 75.0 | S | **S** |
| 38 | The dietitian has a systematic and consistent process of documentation including clinical outcomes relating to nutrition care | 10.0 (9.0-10.0) | 1.4 | 7.2 | 89.9 | S | 9.0 (7.5-10.0) | 2.9 | 14.5 | 79.7 | S | **S** | N/A | | | | | | | | | | |
| 39 | The treating health service has communication and/or shared decision making within the multidisciplinary team about nutrition care | 10.0 (9.0-10.0) | 0.0 | 4.3 | 94.2 | S | 8.0 (6.0-9.0) | 7.2 | 31.9 | 56.5 | M | **M** | 10.0 (9.0-10.0) | 0.0 | 5.0 | 95.0 | S | 8.0 (6.0-9.0) | 6.7 | 21.7 | 71.7 | S | **S** |
| TRAINING AND EDUCATION | | | | | | | | | | | | | | | | | | | | | | | |
| 40 | The treating health service provides training and education support for dietitians about how to deliver and support nutrition care | 10.0 (9.0-10.0) | 0.0 | 10.1 | 88.4 | S | 7.0 (5.0-9.0) | 8.7 | 31.9 | 55.1 | M | M | 10.0 (9.0-10.0) | 0.0 | 6.7 | 91.7 | S | 7.5 (5.0-9.0) | 6.7 | 25.0 | 68.3 | M | **M** |
| 41 | The treating health service ensures nutrition knowledge and skills are maintained and there is limited variation in practice between dietitians providing nutrition care | 10.0 (9.0-10.0) | 0.0 | 5.8 | 92.8 | S | 7.0 (5.0-9.0) | 5.8 | 34.8 | 55.1 | M | M | 10.0 (9.0-10.0) | 0.0 | 1.7 | 98.3 | S | 7.0 (5.0-8.0) | 11.7 | 21.7 | 65.0 | M | **M** |
| 42 | The treating health service provides training and education support for medical, nursing, allied health staff and/or those directly involved in nutrition care | 9.5 (8.0-10.0) | 1.4 | 5.8 | 91.3 | S | 7.0 (5.0-8.0) | 11.6 | 26.1 | 58.0 | M | M | 9.5 (8.0-10.0) | 0.0 | 3.3 | 96.7 | S | 7.0 (6.0-8.0) | 3.3 | 35.0 | 60.0 | M | **M** |
| 43 | The treating health service ensures nutrition knowledge and skills are | 9.0 (8.0-10.0) | 0.0 | 8.7 | 89.9 | S | 6.0 (5.0-7.0) | 15.9 | 49.3 | 29.0 | W | M | 9.0 (8.0-10.0) | 0.0 | 6.7 | 93.3 | S | 6.0 (5.0-7.0) | 8.3 | 55.0 | 35.0 | W | **M** |

| | | | | | | | | | | | | | | | | | | | | | | | |
|---|---|---|---|---|---|---|---|---|---|---|---|---|---|---|---|---|---|---|---|---|---|---|---|
| | maintained and there is limited variation in practice between medical, nursing and/or allied health staff providing and supporting nutrition care | | | | | | | | | | | | | | | | | | | | | | |
| 44 | The treating health service provides training and education support for dietitians/other staff to support the planning, development, implementation, and maintenance of nutrition care into clinical practice | 10.0 (8.0-10.0) | 2.9 | 4.3 | 89.9 | S | 7.0 (5.0-8.0) | 11.6 | 24.6 | 58.0 | M | M | 10.0 (9.0-10.0) | 1.7 | 3.3 | 95.0 | S | 7.0 (5.5-8.0) | 10.0 | 25.0 | 63.3 | M | **M** |
| 45 | The treating health service provides training and education support for support staff, kitchen staff and/or those indirectly involved in nutrition care | 9.0 (8.0-10.0) | 1.4 | 10.1 | 85.5 | S | 6.0 (5.0-8.0) | 11.6 | 39.1 | 44.9 | W | M | 9.0 (8.0-10.0) | 0.0 | 5.0 | 95.0 | S | 7.0 (5.0-8.0) | 10.0 | 31.7 | 56.7 | M | **M** |
| NUTRITION GOVERNANCE AND DATA | | | | | | | | | | | | | | | | | | | | | | | |
| 46 | The treating health service assigns roles and responsibilities for nutrition care | 10.0 (9.0-10.0) | 0.0 | 4.3 | 94.2 | S | 8.0 (6.0-10.0) | 5.8 | 20.3 | 69.6 | M | **M** | 10.0 (9.0-10.0) | 0.0 | 1.7 | 98.3 | S | 8.0 (7.0-10.0) | 1.7 | 13.3 | 83.3 | S | S |
| 47 | The treating health service has governance systems including regular quality improvement and audits of nutrition care practices to support nutrition care | 10.0 (8.0-10.0) | 1.4 | 2.9 | 94.2 | S | 7.5 (6.0-8.0) | 7.2 | 21.7 | 66.7 | M | **M** | 10.0 (9.0-10.0) | 0.0 | 3.3 | 96.7 | S | 8.0 (6.0-8.0) | 3.3 | 25.0 | 70.0 | S | S |
| 48 | The treating health service has relevant nutrition policies, procedures, and guidelines to support nutrition care | 10.0 (9.0-10.0) | 4.3 | 0.0 | 94.2 | S | 9.0 (7.0-10.0) | 5.8 | 13.0 | 76.8 | S | **S** | N/A | | | | | | | | | | |
| 49 | The treating health service collects data to support evidence of potential cost savings and 'return on investment' for nutrition care | 10.0 (8.0-10.0) | 4.3 | 7.2 | 87.0 | S | 6.0 (5.0-8.0) | 11.6 | 37.7 | 46.4 | W | **M** | 9.0 (8.0-10.0) | 1.7 | 10.0 | 88.3 | S | 6.0 (5.0-7.0) | 6.7 | 50.0 | 41.7 | W | **M** |
| PEOPLE WITH CANCER AND CAREGIVER INPUT | | | | | | | | | | | | | | | | | | | | | | | |

| | | | | | | | | | | | | | | | | | | | | | | |
|---|---|---|---|---|---|---|---|---|---|---|---|---|---|---|---|---|---|---|---|---|---|---|
| 50 | People with cancer and their carers have input and feedback into the design and evaluation of nutrition care and services | 10.0 (9.0-10.0) | 0.0 | 4.3 | 92.8 | S | 7.0 (6.0-8.5) | 4.3 | 31.9 | 60.9 | M | **M** | 10.0 (9.0-10.0) | 1.7 | 3.3 | 95.0 | S | 7.0 (6.0-8.0) | 6.7 | 28.3 | 63.3 | M | **M** |
| 51 | People with cancer and their carers have high awareness of what nutrition services exist and the availability of dietitians within their treating health service following a cancer diagnosis | 10.0 (9.0-10.0) | 1.4 | 4.3 | 92.8 | S | 8.0 (5.0-8.5) | 10.1 | 18.8 | 68.1 | M | **M** | 10.0 (9.0-10.0) | 1.7 | 1.7 | 96.7 | S | 8.0 (6.0-9.0) | 5.0 | 26.7 | 66.7 | M | **M** |
| 52 | People with cancer and their carers have high awareness of what primary care and community-based nutrition services exist and the availability of dietitians outside of their treating health service following a cancer diagnosis | 9.5 (8.0-10.0) | 1.4 | 8.7 | 88.4 | S | 7.0 (5.0-8.0) | 11.6 | 33.3 | 52.2 | M | **M** | 9.0 (8.0-10.0) | 3.3 | 0.0 | 96.7 | S | 7.0 (5.0-8.0) | 8.3 | 33.3 | 56.7 | M | **M** |

[a]Ratings were on an 11-point unipolar Likert scale from '0' (not at all important/feasible) to '10' (extremely important/feasible), or unable to rate; [b]rating low = Likert score <4: rating moderate = Likert score 4-<7: rating high = Likert score ≥7-10: unable to rate or missing data not reported under any rating; ; [c]*S* = Strong consensus i.e., ≥70% of participants indicated a highly rated response for both important and feasibility (cell coloured green): *M* = Medium consensus i.e., ≥70% of participants indicated a highly rated response for one of importance or feasibility and between 50% and <70% indicated a highly rated response for the alternate of either importance or feasibility (cell coloured yellow): *W* = Weak consensus i.e., <50% of participants indicated a highly rated response for both importance and feasibility or one <50% and the alternate <70% (cell coloured orange).

Abbreviations: R1=Round1; R2=Round 2; IQR=Interquartile range; L=low; M=moderate; H=high; Consensus=cons.
